# Mitochondrial succinate transport is required for cardiac ischaemia/reperfusion injury

**DOI:** 10.1093/cvr/cvag031

**Published:** 2026-01-28

**Authors:** Laura Pala, María Torres-López, Stuart T Caldwell, Joyce Valadares, Emily M Smith, Katherine L Hammond, Olga Sauchanka, Jiro Abe, Thomas Krieg, Richard C Hartley, Michael P Murphy, Hiran A Prag

**Affiliations:** School of Chemistry, University of Glasgow, Glasgow G12 8QQ, UK; Institute of Biomedicine of Seville (IBiS), Hospital ‘Universitario Virgen del Rocío’/CSIC/University of Seville, Seville 41013, Spain; Department of Medical Physiology and Biophysics, School of Medicine, University of Seville, Seville 41009, Spain; School of Chemistry, University of Glasgow, Glasgow G12 8QQ, UK; MRC Mitochondrial Biology Unit, University of Cambridge, Cambridge CB2 0XY, UK; MRC Mitochondrial Biology Unit, University of Cambridge, Cambridge CB2 0XY, UK; MRC Mitochondrial Biology Unit, University of Cambridge, Cambridge CB2 0XY, UK; Department of Medicine, University of Cambridge, Cambridge CB2 0QQ, UK; MRC Mitochondrial Biology Unit, University of Cambridge, Cambridge CB2 0XY, UK; Department of Medicine, University of Cambridge, Cambridge CB2 0QQ, UK; School of Chemistry, University of Glasgow, Glasgow G12 8QQ, UK; MRC Mitochondrial Biology Unit, University of Cambridge, Cambridge CB2 0XY, UK; Department of Medicine, University of Cambridge, Cambridge CB2 0QQ, UK; MRC Mitochondrial Biology Unit, University of Cambridge, Cambridge CB2 0XY, UK; Department of Medicine, University of Cambridge, Cambridge CB2 0QQ, UK

**Keywords:** Succinate, Ischaemia/reperfusion injury, Mitochondrial dicarboxylate carrier, SLC25A10, Mitochondrial transport, Myocardial infarction, Butylmalonate

## Abstract

**Aims:**

Succinate accumulates significantly during myocardial ischaemia, and its rapid oxidation upon reperfusion is a critical driver of ischaemia/reperfusion (I/R) injury. The transport of succinate across the mitochondrial inner membrane, particularly by the dicarboxylate carrier (DIC; SLC25A10), is hypothesized to play a crucial role in mediating these pathological succinate dynamics. However, tools to test this hypothesis by modulating mitochondrial succinate transport in biological systems are lacking.

**Methods and results:**

C57BL/6J mice, isolated Wistar Rat heart mitochondria, bovine heart mitochondrial membranes, C2C12 mouse myoblasts, and primary adult mouse cardiomyocytes were used as *in vitro* and *in vivo* models. Butylmalonate prodrugs were synthesized and tested. Isolated mitochondria were used to assess succinate-dependent respiration and reactive oxygen species (ROS) production. Cells were treated with succinate dehydrogenase (SDH) inhibitors or exposed to anoxia and butylmalonate esters. Mouse hearts were subjected to *in vivo* left anterior descending coronary artery ligation. Succinate and butylmalonate levels were measured by targeted liquid chromatography-tandem mass spectrometry, and infarct size by TTC (23,5-triphenyl-2H-tetrazolium chloride) staining. Knockdown of DIC, but not of the oxoglutarate carrier OGC, in C2C12 cells prevented succinate accumulation by SDH inhibition and anoxia. The only extant DIC inhibitor butylmalonate, is limited by poor cell permeability. We synthesized diacetoxymethyl butylmalonate (DAB), which efficiently delivers butylmalonate intramitochondrially in isolated heart mitochondria and cells. DAB inhibited succinate-dependent respiration and ROS production. DAB prevented succinate accumulation in cells treated with SDH inhibitors. DAB delivered butylmalonate to cardiac mitochondria when administered to mice *in vivo* and reduced infarct size by perturbing mitochondrial succinate transport.

**Conclusion:**

The DIC is a key node in the cellular distribution of succinate, controlling its transport between mitochondria and the cytosol. These findings highlight the potential of DIC as a promising therapeutic target for conditions where succinate elevation contributes to pathogenesis, such as cardiac I/R injury.

## Graphical Abstract

**Graphical Abstract:**
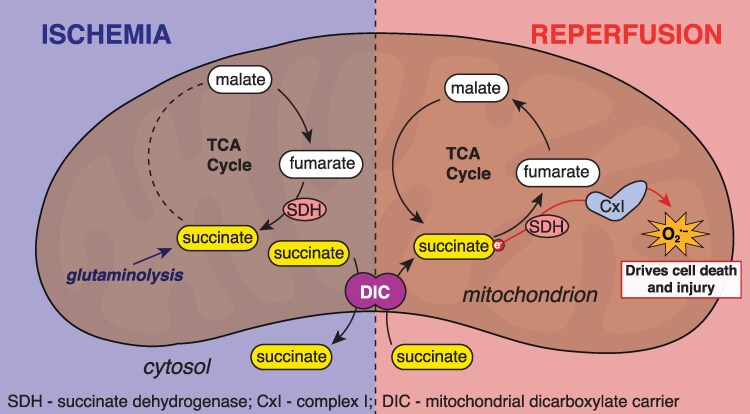

**Time of primary review: 43 days**

**See the editorial comment for this article ‘Unravelling mitochondrial succinate transport during I/R and devising a novel transport inhibitor show promise for targeting I/R injury, but will efficacy remain under more human-like conditions present during I/R?’, by Q. Wang**  ***et al*****., https://doi.org/10.1093/cvr/cvag059.**

## 1. Introduction

Charged metabolites and ions are selectively permeable across the mitochondrial inner membrane, a requirement to maintain a proton electrochemical potential gradient to drive oxidative phosphorylation.^1^ Consequently, the flux of metabolites between the mitochondrial matrix and cytosol depends on the action of multiple transporters, working in tandem to maintain homeostasis.^1,2^ The mitochondrial carrier SLC25 family of 53 transporters in humans is mainly involved in transporting a range of metabolites and ions between the matrix and cytosol, essential for maintaining mitochondrial and cellular function.^1,2^ These SLC25 family members reside in the mitochondrial inner membrane, predominantly acting as structural and functional monomers, with a single central substrate-binding site.^2^ Two salt-bridge networks, a matrix glutamine brace and a cytosolic tyrosine brace, regulate access of substrates to the binding site from either side of the membrane.^3,4^ While the specific transport mechanism of each of the mitochondrial carriers is yet to be determined, recent evidence suggests a ping-pong kinetic mechanism of substrate transfer across the inner membrane.^5^ The carrier, now in its inward-facing conformation, binds the exchange substrate from the matrix. A reciprocal conformational change facilitates translocation and dissociation of the exchange substrate into the cytoplasm-facing side, completing the exchange cycle of the mitochondrial metabolite.^1–4^ Despite being critical transport processes, as evidenced by the often severe phenotypes of patients harbouring mutations,^6–8^ their contribution to general physiology and pathology is underappreciated. Furthermore, around one-third of the SLC25 family are still orphan transporters.^2^

**Figure 1:**
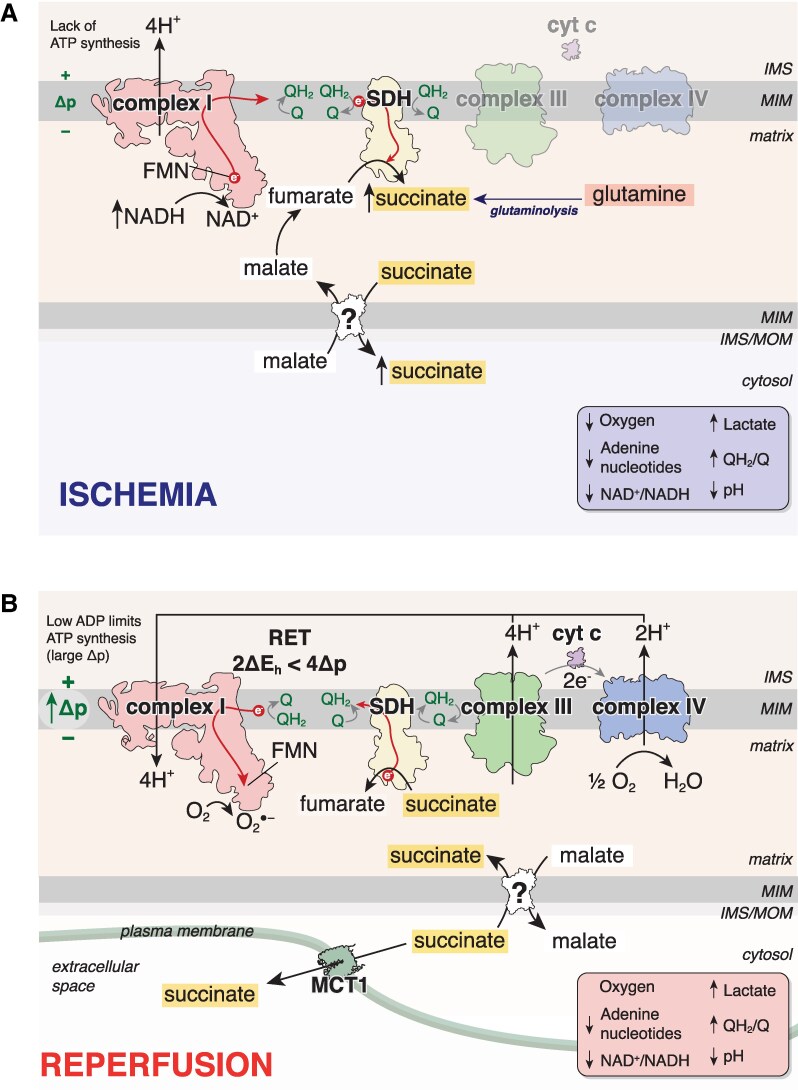
Schematic of targeting succinate metabolism in IR injury. (*A*) Under ischaemic conditions, succinate levels are dramatically elevated. This is due to the reversal of SDH and a contribution from glutaminolysis. Succinate has been proposed to enter the cytosol in exchange for malate to sustain succinate production and thus the cytosol acts as an electron sink, enabling large succinate accumulation. (*B*) Upon reperfusion, a proportion of cytosolic succinate exits the cell via the MCT1. The remaining succinate must re-enter the mitochondria to be subsequently oxidized by SDH, thereby driving ROS production by RET. MIM, mitochondrial inner membrane; MOM, mitochondrial outer membrane; IMS, intermembrane space; SDH, succinate dehydrogenase; FMN, flavin mononucleotide; Δp, proton motive force; QH_2_, ubiquinol; Q, ubiquinone, MCT1, monocarboxylate transporter 1; cyt c, cytochrome c; $O_{2}^{\cdot -}$ superoxide.

Recently, the tricarboxylic acid (TCA) cycle metabolite succinate has emerged as a key player in various (patho)physiological processes, such as its role in driving ischaemia/reperfusion (I/R) injury^9–11^ and inflammation.^12–14^ In myocardial infarction (MI), when a coronary blood vessel is occluded, and underlying tissue becomes ischaemic,^15^ there is an up to 14-fold accumulation of succinate in ischaemic tissue,^9,11^ reaching millimolar concentrations. Succinate accumulation is thought to be driven by succinate dehydrogenase (SDH) reversal or canonical TCA cycle activity via glutaminolysis, however, there remains conflicting evidence as to the mechanisms involved, depending on ischaemia time and experimental model used (*Figure 1*).^9,16,17^ To sustain succinate accumulation at this level, it is postulated that an enabling step is succinate transport into the cytosol, in exchange for other substrates, most notably malate.^16,18^ This metabolite exchange not only enables the cytosol to act as an electron sink during ischaemia by storing succinate, but the malate transported into the mitochondrial matrix can further feed partial TCA cycle reversal via dehydration to fumarate and reduction to succinate at SDH.^16,18^ Of particular interest to mitochondrial succinate and malate transport are SLC25A10 and SLC25A11, which are the mitochondrial dicarboxylate (DIC) and oxoglutarate (OGC) carriers, respectively.^19–21^ DIC and OGC overlap in their substrate specificity, with both carriers capable of transporting malate and succinate at least under *in vitro* conditions.^19,21,22^ Upon reperfusion of ischaemic tissues, the accumulated succinate is rapidly oxidized by SDH, which may drive superoxide production by reverse electron transport (RET) at mitochondrial complex I.^9,23,24^ This superoxide burst is thought to set in train several downstream events, which can culminate in cell death and tissue damage.^9,23,25–27^ Modulating succinate metabolism by inhibiting SDH elicits cardioprotection in both rodent and pig models of MI.^9,27–31^ Therefore, by altering the flow of succinate between compartments, DIC and OGC may be novel therapeutic targets when succinate is implicated in pathogenesis.

While butylmalonate is an effective inhibitor of the mitochondrial DIC, its use has been limited to inhibiting metabolite transport in isolated mitochondria, transporter or vesicle studies.^19,21,32^ Due to the poor membrane permeability of butylmalonate, simple butylmalonate ester prodrugs, such as diethyl butylmalonate, have been used in *in vitro* cell studies, however, we have found that these approaches deliver insufficient butylmalonate to be biologically effective, particularly during acute treatment. Therefore, suitable tools to transiently inhibit DIC and OGC *in vitro* and *in vivo* are lacking. To overcome this, we used a tuned prodrug approach to develop a means of delivering butylmalonate within cells *in vitro* and *in vivo*. We found that diacetoxymethyl butylmalonate (DAB) delivered butylmalonate intracellularly, with a proportion of the butylmalonate generated within the mitochondrial matrix. We found that the perturbation of succinate metabolism by DAB was similar to that found by knockdown (KD) of DIC, but not of OGC, suggesting that DIC is the main succinate carrier and the primary target of butylmalonate. Inhibiting mitochondrial succinate transport with DAB was protective against cardiac I/R injury in an MI model, demonstrating the importance of succinate transport by DIC in pathology.

## 2. Methods

### 2.1 Cell maintenance

C2C12 murine myoblasts were obtained from American Type Culture Collection (ATCC) and maintained at 37°C, 5% CO_2_ and 100% humidity. Cell media was changed every 2–3 days with passaging at <80% confluency, seeded at a density less than 5 × 10^3^ viable cells/cm^2^ with a typical subcultivation ratio of 1:5–1:10 in DMEM media (4.5 g/L glucose, 1 mM sodium pyruvate, 2 mM Glutamax, 1.5 g/L sodium bicarbonate) with 10% Fetal Bovine Serum (FBS). Primary adult murine cardiomyocytes were isolated, maintained and used as described in detail previously.^11,26^

### 2.2 Animals

Experiments were performed following the UK Animals (Scientific Procedures) Act of 1986, Guide for the Care and Use of Laboratory Animals, published by the US National Institutes of Health (NIH Publication No. 85-23, revised 1996) and the University of Cambridge Animal Welfare Policy under project licence PP4344323, reviewed by the University of Cambridge Animal Welfare Ethical Review Board. Wistar rats (female, 10–12 weeks) and C57BL/6J mice (male, 8–12 weeks) were ordered from Charles River Laboratories UK (Margate, UK) and maintained on chow and water *ad libitum*. Only male mice were used in this study as this provided the best comparison to previous work on succinate as a driver of I/R injury. For the acute murine MI model, mice were culled via exsanguination by division of the abdominal IVC. For isolation of adult cardiomyocytes, mice were culled by cervical dislocation (no anaesthetic used). For isolation of rat heart mitochondria (RHM), rats were culled by cervical dislocation (no anaesthetic used).

### 2.3 LC-MS/MS analysis of metabolites

Butylmalonate, succinate and malonate were quantified using LC-MS/MS as described in detail previously.^27^ Briefly, 1–5 µL sample was injected onto a SeQuant ZIC-HILIC column (3.5 μm, 100 Å, 150 × 2.1 mm, 30°C column temperature; Merck Millipore, UK) with a ZIC-HILIC guard column (200 Å, 1 × 5 mm) and separated using a flow rate of 0.2 mL/min with mobile phases of (i) 10 mM ammonium bicarbonate and (ii) 100% acetonitrile using a Nexera UHPLC system (Shimadzu, UK). Spectra were detected using an LCMS-8060 mass spectrometer (Shimadzu, UK) and acquired using LabSolutions software (Shimadzu, UK). Compounds were quantified via interpolation of a standard curve compared with internal standards (1 nmol [^13^C_3_]-malonate for butylmalonate and malonate and [^13^C_4_]-succinate for succinate).

### 2.4 Incubation of cells with butylmalonate ester prodrugs

C2C12 cells were plated in 6-well plates (500 000 cells/well) and adhered overnight. The following day, the medium was replaced with Krebs buffer (116 mM NaCl, 4.7 mM KCl 1.2 mM MgSO_4_.7H_2_O, 25 mM HEPES, 1.4 mM CaCl_2_, 11 mM glucose; pH 7.4, 37°C) containing disodium butylmalonate, butylmalonate ester prodrugs or 0.1% DMSO (control) and incubated for 15–240 min at 37°C prior to extracting. Parallel plates were incubated under the same conditions and used to measure protein levels by BCA assay (Thermo Fisher Scientific, UK). After incubation, cells were used for rapid mitochondrial preparation or extracted for LC-MS/MS.

### 2.5 *In vivo* delivery of DAB

DAB (16 mg/kg) was administered to C57BL/6J mice via a 100 μL tail vein bolus injection or infusion over 5 min in [0.9% saline, 1% DMSO and 30% β-cyclodextrin (Captisol, Ligand, USA)]. For most studies, mice were culled 5 min after bolus injection and mitochondria rapidly isolated from the heart tissue using the method previously described.^33^ When DAB was used in the MI model, the 100 µL was infused over 5 min, either starting 2.5 min before ischaemia induction or 2.5 min before reperfusion.

### 2.6 *In vivo* left anterior descending coronary artery occlusion murine MI model

The left anterior descending (LAD) coronary artery was ligated to induce MI as described in detail previously.^34^ Briefly, mice were anesthetized using sodium pentobarbital (70 mg/kg intraperitoneally), endotracheally intubated and ventilated with 3 cm H_2_O positive end-expiratory pressure. Core temperature was maintained at 37°C using a rectal thermometer-controlled heatpad (TCAT-2LV, Physitemp, USA). Ventilation frequency was maintained at 110 breaths/min, with tidal volume between 125 and 150 μL. Mice were subjected to 30 min of ischaemia and 120 min of reperfusion; for infarct analysis, after reperfusion, hearts were stained with Evans Blue and 2% triphenyltetrazolium chloride (TTC) and blindly analysed by an independent researcher. Investigative drugs were administered via the tail vein in a maximum volume of 100 µL.

### 2.7 Rapid mitochondrial isolation

The rapid mitochondria prep from cells was developed based on a previous method for tissue isolation,^33^ which was used for isolating mitochondria from tissue for mass spectrometry from tissues in this manuscript. Cells were resuspended in 300 µL hypotonic homogenization buffer (50 mM HEPES sodium, 1 mM EDTA, 1 mg/mL BSA, 1 mg/mL digitonin; pH 7.4, 4°C) and homogenized rapidly by 30 strokes of a tight-fitting pestle in a 2 mL dounce homogeniser. The homogenate was layered on top of 500 µL 38:62 silicone oil:dioctyl phthalate mix with 100 µL isotonic buffer (300 mM sucrose, 50 mM HEPES sodium, 1 mM EDTA, 1 mg/mL BSA; pH 7.4, 4°C) underneath in a 1.5 mL microcentrifuge tube. The tube was centrifuged using a spin-out rotor (9727*×g*, 1 min, 4°C). The resulting pellet was isolated mitochondria and the cytosol remained as the top layer. The cytosol was subsequently centrifuged (17 000×g, 5 min, 4°C) to remove other membraned organelles.

### 2.8 *In vitro* ischaemia

Primary cardiomyocytes were isolated and *in vitro* ischaemia was carried out as described previously.^26^ C2C12 cells (500 000 cells/dish) or primary adult cardiomyocytes (100 000 cardiomyocytes/dish) were plated in 60 mm glass dishes coated with 10 µg/mL poly-D-lysine (C2C12s) or 0.1 mg/mL laminin (cardiomyocytes), and incubated for 24 h with DMEM or M199. Cells were subsequently incubated in Tyrode’s buffer (137 mM NaCl, 5.4 mM KCl, 0.4 mM MgCl_2_, 10 mM HEPES, 5 mM glucose, 1 mM CaCl_2_, pH 7.4) supplemented with 10 µM MCT1 inhibitor AR-C141990 (Tocris, Bioscience). Cells were placed in an anaerobic chamber (<0.5 ppm O_2_; Belle Technologies, UK) on a 37°C heatblock for 1 h before extraction or subsequent experiments. For reperfusion, medium was replaced with MCT1 inhibitor-free Tyrode’s buffer and then cells were removed from the chamber.

### 2.9 Reverse transfection siRNA knockdown

Reverse transfection was carried out according to the Silencer Select siRNA manufacturer’s instructions and optimized for concentration. 500 µL Optimem (Gibco, UK) were added to wells in a 6-well plate, before adding 7.5 µL Lipofectamine RNAiMAX and 5 pmol Silencer Select siRNA (slc25a10: s211655, slc25a11: s85784, Silencer select negative control No. 1), briefly vortexing the plate and incubating for 15 min. 100 000 C2C12 cells were added to the wells and final volume adjusted to 2 mL before the plate was briefly swirled to mix contents. Cells were incubated for 72 h before use in subsequent experiments.

### 2.10 RT-qPCR

RNA was extracted from 6-well plates using a PureLink RNA Mini kit (Invitrogen) according to the manufacturer's instructions, eluted in 50 µL RNase-free water and concentration measured using a Nanodrop spectrophotometer. cDNA was synthesized from 1 µg RNA using a High-Capacity cDNA Reverse Transcription Kit (Applied Biosystems) according to the manufacturer’s instructions before diluting the cDNA 1:10 with water. qPCR was carried out with PowerUp SYBR Green Master Mix (Applied Biosystems) in fast cycling mode using a 96-well QuantStudio 3 Real-Time PCR system (Thermo Fisher Scientific). Primers used for qPCR are in *Table 1*.

**Table 1:** Primers for RT-qPCR

Gene	FW (5′-3′)	RV (5′-3′)
*ACTB* (M)	CATTGCTGACAGGATGCAGAAGG	TGCTGGAAGGTGGACAGTGAGG
*SLC25A10* (M)	CGAATGACTGGATTGGCACTGC	TGGTCTCGTAGATTGCGAACCG
*SLC25A11* (M)	CTCGTTACCACTGCTGCTTCCA	ATAGCGGACGACTTTCAGCAGC

### 2.11 Statistical analysis, randomization, and blinding

All data in figures are presented as mean ± SEM, unless stated otherwise in the figure legend. Data were assessed for normality using the Shapiro–Wilk test. Statistical analysis was performed using two-tailed Student’s *t*-test, Kruskal–Wallis, one or two-way ANOVA with the suitable *post hoc* correction for multiple comparisons described in the figure legend. A *P*-value of <0.05 was considered significant. Statistics were calculated in the Prism 10.0 software (GraphPad Software Inc., USA). Randomization and blinding were carried out where possible: mass spectrometry samples were randomized and analysed blindly; infarct size measurements were carried out in a randomized and blinded fashion by independent investigators.

## 3. Results

### 3.1 Mitochondrial dicarboxylate carrier expression affects succinate metabolism

**Figure 2:**
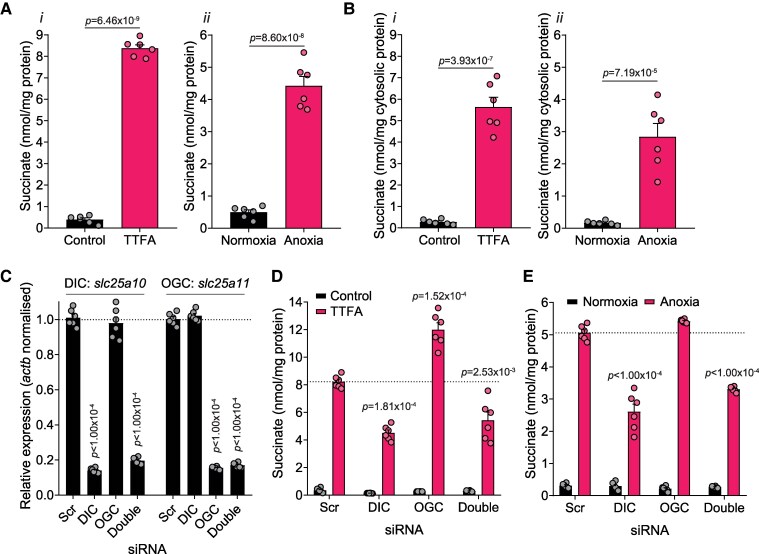
Mitochondrial transport knockdown affects succinate accumulation. (*A*) C2C12 myoblasts (500 000/well) were treated with (i) 500 µM TTFA or (ii) anoxia (<0.5 ppm O_2_) for 1 h and whole cell succinate levels measured (mean ± SEM of *n* = 6 independent experiments). (*B*) Cells treated as in (*A*) were subsequently rapidly fractionated and succinate in cytosol measured (mean ± SEM of *n* = 6 independent experiments). (*C*) RT-qPCR of *slc25a10* and *slc25a11* after siRNA knockdown of DIC, OGC, or scrambled (Scr) control in C2C12s (mean ± SEM of *n* = 6 independent experiments). (*D*) C2C12s after siRNA knockdown of DIC, OGC, or scrambled (Scr) control were treated with 500 µM TTFA for 1 h and whole cell succinate measured (mean ± SEM of *n* = 6 independent experiments, dashed line represents TTFA-treated Scr cells level). (*E*) C2C12 s after siRNA knockdown of DIC, OGC, both or scrambled (Scr) control were exposed to anoxia for 1 h and whole cell succinate measured (mean ± SEM of *n* = 6 independent experiments, dashed line represents anoxia-treated Scr cells level). Statistical significance was assessed by unpaired, two-tailed Student’s *t*-test (*A* and *B*) or one-way ANOVA with Dunnett’s correction for multiple comparisons [*C*–*E*, all compared with scrambled (Scr) siRNA control].

As mitochondrial succinate transport is suggested to be an important facet of various (patho)physiologies including I/R injury, we first examined whether the expression levels of mitochondrial transporters affect cellular succinate levels. To assess this, we used C2C12 mouse myoblasts and initially characterized two established modes of inducing succinate accumulation: SDH inhibition by thenoyltrifluoroacetone (TTFA) or exposing cells to anoxia.^9,26,35^ SDH inhibition or exposure to anoxia led to substantial succinate accumulation compared with untreated or normoxic controls (*Figure 2A*). To understand whether this accumulated succinate is partially stored within the cytosol or solely in the mitochondrial matrix, we sought to measure the cytosolic succinate pool, free of mitochondria. For this, we developed a rapid mitochondrial isolation technique for cultured cells, which utilizes the cholesterol-selective membrane permeabilization by digitonin followed by centrifugation through a layer of oil to rapidly isolate mitochondria and cytosol fractions,^33^ thereby separating their metabolite pools while minimizing redistribution (see Supplementary material online, *Figure S1*). Succinate levels in the cytosol from cells either treated with TTFA or exposed to anoxia were significantly elevated as quantified by LC-MS/MS (*Figure 2B*), confirming that succinate transport from mitochondria to the cytosol, driven by the elevated mitochondrial succinate levels (see Supplementary material online, *Figure S1D*), is a key aspect of succinate accumulation. As both DIC and OGC can transport succinate across the mitochondrial inner membrane, we next used siRNA to knockdown (KD) expression of DIC and OGC, either individually or together, to assess their effects on succinate accumulation. Both OGC and DIC siRNA led to KD efficiencies >80% in C2C12 cells as measured by RT-qPCR (*Figure 2C*). We next assessed whether these KDs affected succinate accumulation by either SDH inhibition or anoxia. We found that DIC KD decreased succinate accumulation compared with control after either TTFA treatment or anoxia (*Figure 2D* and *E*). Intriguingly, OGC KD led to elevated succinate accumulation upon SDH inhibition compared with control, though anoxic succinate levels remained comparable to control (*Figure 2D* and *E*). Double knockdown of both DIC and OGC mirrored DIC knockdown for both SDH inhibition and anoxia (*Figure 2D* and *E*). As decreasing the expression of the DIC perturbed succinate accumulation with SDH inhibition and anoxia, this suggested inhibiting the transport activity of DIC as a useful strategy to target succinate accumulation and oxidation in I/R injury.

### 3.2 Butylmalonate blocks succinate transport across the mitochondrial inner membrane

**Figure 3:**
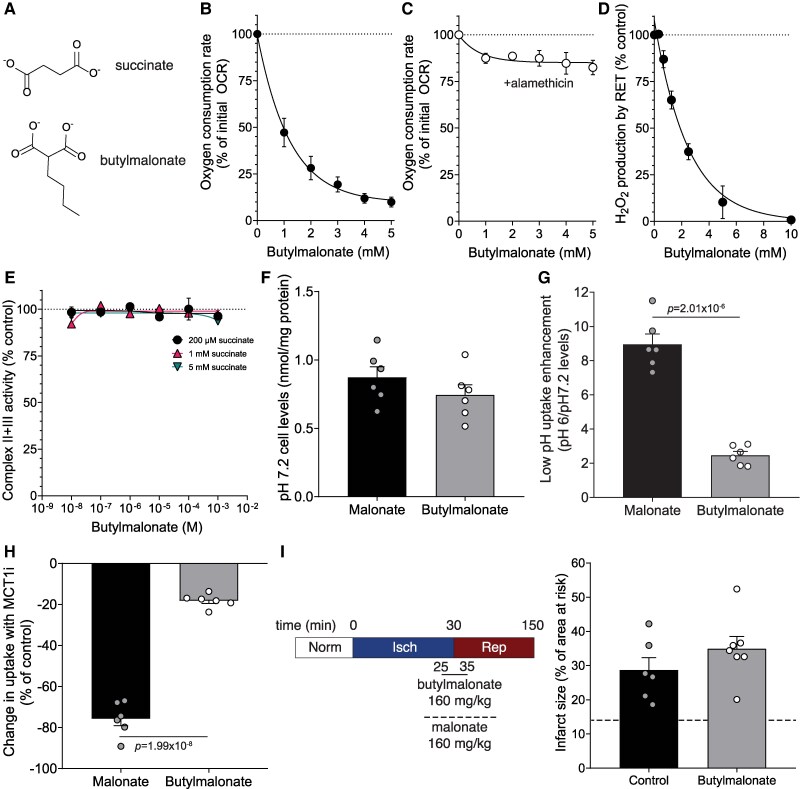
Characterizing DIC inhibition by butylmalonate. (*A*) Structures of succinate and butylmalonate. (*B* and *C*) Rat heart mitochondria (1 mg/mL) were incubated with 1 mM succinate, 1 µM FCCP and 4 µg/mL rotenone and oxygen consumption measured (*C*) oxygen consumption was measured as in (*B*) but in the presence of alamethicin (40 µg/mL) (mean ± SEM, *n* = 4 biological replicates). (*D*) Rat heart mitochondria were incubated with succinate (5 mM) ± butylmalonate and H_2_O_2_ production measured by resorufin fluorescence (mean ± SEM, *n* = 4 biological replicates). (*E*) Complex II + III activity in bovine heart mitochondrial membranes incubated with succinate and butylmalonate (mean ± SEM, *n* = 4 independent experiments). (*F*) C2C12 cells were treated with 5 mM malonate or butylmalonate at pH 7.2 for 30 min and uptake measured by LC-MS/MS (mean ± SEM levels of compound, *n* = 6 biological replicates). (*G*) C2C12 cells were treated as (*F*) at pH 7.2 and pH 6 (mean ± SEM levels of compound at pH 6/levels of compound at pH 7.2, *n* = 6 biological replicates). (*H*) Reduction in uptake of malonate or butylmalonate in cells treated as in (*F*) but in the presence of MCT1 inhibitor (MCT1i) 10 µM AZD3965 (mean ± SEM, *n* = 6 independent experiments). (*I*) *In vivo* infarct size after 30 min LAD ligation ischaemia and 2 h reperfusion with control (saline) or disodium butylmalonate (160 mg/kg) infused upon reperfusion (mean ± SEM, *n* = 6 (control) or 7 (butylmalonate) biological replicates). Dashed line is representative infarct size with 160 mg/kg disodium malonate. Statistical significance was assessed by unpaired, two-tailed Student’s *t*-test (*F*,*G*,*H*).

As KD of DIC, but not OGC, significantly decreases succinate accumulation, we focused on the DIC as a target for pharmacological development. Butylmalonate (*Figure 3A*) has historically been used to inhibit the DIC in mitochondria *in vitro.*^21,32^ Despite being used in cell and *ex vivo* experiments,^36–38^ butylmalonate entry into cells at appropriate concentrations to inhibit the DIC was seldom confirmed; thus, off-target effects may have contributed. We confirmed the utility of butylmalonate in isolated rat heart mitochondria and found it inhibited succinate-dependent respiration, in a dose-dependent manner (*Figure 3B*). When the mitochondrial inner membrane was permeabilized with the pore-former alamethicin, the inhibitory effects of butylmalonate on respiration were lost (*Figure 3C*), confirming its action via DIC. Butylmalonate also dose-dependently suppressed reactive oxygen species (ROS) production by succinate-driven RET in isolated heart mitochondria (*Figure 3D*). Inhibiting SDH with malonate reduces succinate-driven RET-ROS upon reperfusion; however, butylmalonate did not inhibit SDH activity (*Figure 3E*). Thus, the effects of butylmalonate on respiration and RET-ROS production driven by exogenous succinate are solely by restricting succinate entry into mitochondria.

### 3.3 Butylmalonate cellular uptake is limited

We next assessed whether butylmalonate itself could also be an effective DIC inhibitor in cells. Incubating C2C12 mouse myoblasts with 5 mM butylmalonate at pH 7.4 led to low levels of uptake into the cells, comparable to those of malonate when incubated at the same concentration (*Figure 3F*). Malonate uptake into cells is significantly enhanced (∼9-fold) at pH 6 compared with 7.4 (*Figure 3G*), because mono-protonated malonate is a good substrate for the monocarboxylate transporter 1 (MCT1).^27,31^ In contrast, butylmalonate uptake into cells was only enhanced ∼2-fold at pH 6 and this uptake was unaffected by the MCT1 inhibitor AZD3965 (*Figure 3H*). Using the LAD coronary artery ligation model of cardiac I/R injury, we found that butylmalonate was not cardioprotective *in vivo* when administered upon reperfusion, while malonate was because its uptake via the MCT1 is enhanced by the low pH of ischaemia^27^ (*Figure 3I* and Supplementary material online, *Figure S2*). Thus, despite butylmalonate inhibiting the DIC *in vitro*, its lack of uptake across the plasma membrane even at low pH prevents its inhibition of the DIC in cells or *in vivo*. Therefore, we set out to enhance butylmalonate delivery across the plasma membrane to better explore the impact of targeting the DIC *in vitro* and *in vivo*.

### 3.4 Tuned butylmalonate prodrugs rapidly deliver butylmalonate within cells

**Figure 4:**
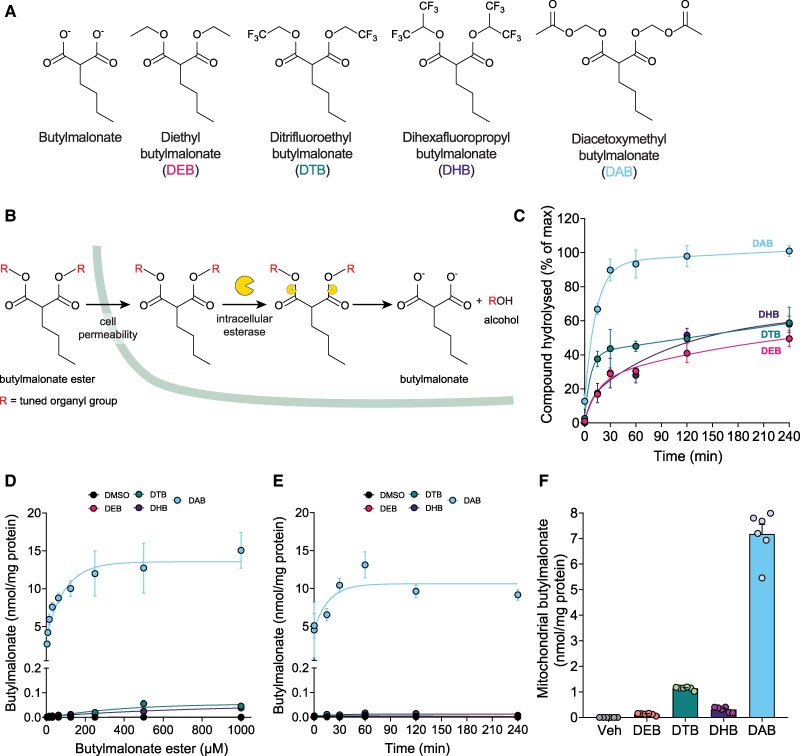
Tuned butylmalonate esters. (*A*) Structures of butylmalonate ester prodrugs. (*B*) Schematic of proposed prodrug approach to butylmalonate delivery. (*C*) 200 µM butylmalonate ester prodrugs were incubated with porcine liver esterase (PLE; 1 mg/mL) and butylmalonate released measured by LC-MS/MS (mean ± SEM of butylmalonate released compared with complete hydrolysis, *n* = 3 independent experiments). (*D*) C2C12 cells (500 000 cells/well) were incubated with different concentrations of butylmalonate esters or 0.1% DMSO for 30 min before butylmalonate was measured by LC-MS/MS (mean ± SEM, *n* = 6 biological replicates). (*E*) C2C12 cells (500 000 cells/well) were incubated with 250 µM butylmalonate esters or 0.1% DMSO for varying time before butylmalonate was measured by LC-MS/MS (mean ± SEM, *n* = 6 biological replicates). (*F*) Isolated rat heart mitochondria (1 mg/mL) were incubated with 250 µM butylmalonate esters or 0.1% DMSO vehicle control (Veh) (15 min) and intramitochondrial butylmalonate measured by LC-MS/MS (mean ± SEM, *n* = 6 biological replicates).

To counteract its poor cell uptake, we synthesized a range of hydrophobic butylmalonate ester prodrugs designed to be membrane-permeable, while also undergoing rapid enzymatic hydrolysis to release butylmalonate intracellularly (*Figure 4A* and *B* and Supplementary material online, Chemical Synthesis). These included simple alkyl esters [diethylbutylmalonate (DEB)], esters with enhanced hydrolysis due to electron-withdrawing, fluorine-containing alkyl groups [ditrifluoroethyl butylmalonate (DTB) and dihexafluoropropyl butylmalonate (DHB)], or by incorporation of the esterase-cleavable acetoxymethyl group [diacetoxymethyl butylmalonate (DAB)]. Using the model esterase porcine liver esterase (PLE)^30,39^ we found that only DAB and MHB were completely hydrolysed within 4 h *in vitro* to release butylmalonate, assessed by LC-MS/MS (*Figure 4C*). We next incubated C2C12 cells with varying concentrations of butylmalonate esters for 30 min before measuring intracellular butylmalonate levels. We found that DAB led to >30-fold higher levels of intracellular butylmalonate compared with any of the other compounds (*Figure 4D*). Butylmalonate levels plateaued with DAB concentrations of 250 µM and above, reaching ∼10-fold higher levels than cells incubated with 5 mM butylmalonate alone (*Figures 3F* and *4D*). Furthermore, even after 4 h incubation of cells with the 250 µM butylmalonate esters, only DAB led to significant intracellular butylmalonate levels that were retained within the cell (*Figure 4E*). Maximal levels of butylmalonate were achieved after 60 min incubation time, with levels plateauing with further incubation time (*Figure 4E*). Importantly, succinate levels under aerobic conditions were unchanged by any of the butylmalonate esters, suggesting a lack of off-target effects on succinate metabolism (see Supplementary material online, *Figure S3A* and *B*). To understand whether butylmalonate esters may also deliver butylmalonate inside mitochondria to the matrix, we next incubated isolated heart mitochondria for 15 min with butylmalonate esters before measuring intramitochondrial butylmalonate levels. We found DAB led to ∼7 nmol/mg protein butylmalonate delivered within mitochondria, with ∼1 nmol/mg protein delivered by DHB and DTB and negligible delivery by DEB (*Figure 4F*). Overall, these results suggested that DAB was the most appropriate compound for further characterization as a DIC inhibitor *in vitro* and *in vivo*.

### 3.5 DAB modulates succinate metabolism *in vitro*

**Figure 5:**
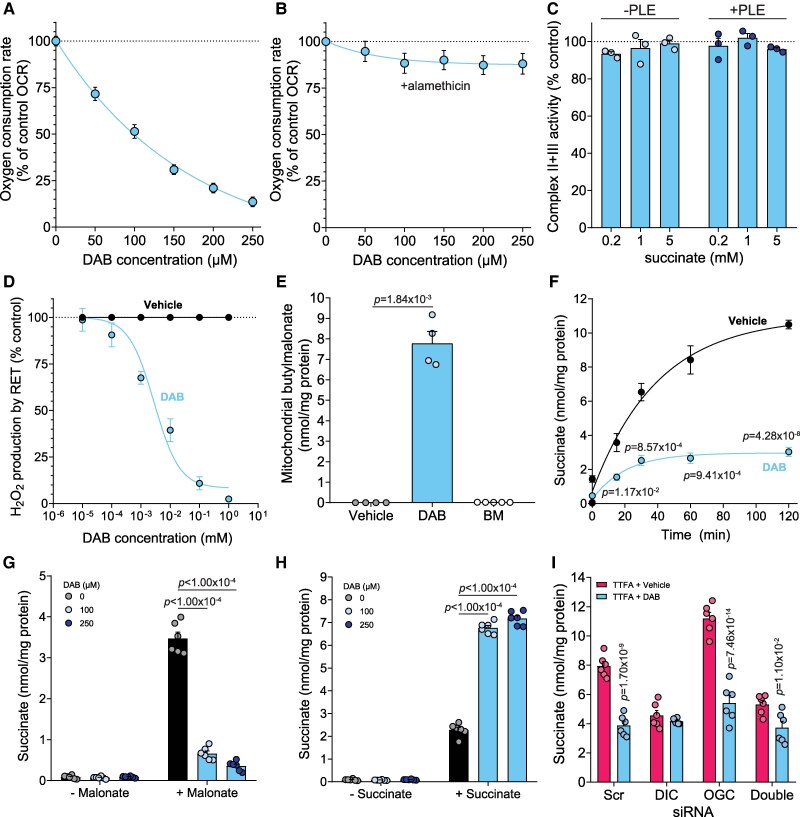
DAB inhibits DIC *in vitro*. (*A* and *B*) Rat heart mitochondria (1 mg/mL) were incubated with 1 mM succinate, 1 µM FCCP, 4 µg/mL rotenone, and DAB and oxygen consumption measured. (*B*) Oxygen consumption was measured as in (*A*) but in the presence of alamethicin (40 µg/mL) (mean ± SEM, *n* = 6 biological replicates). (*C*) Complex II + III activity in bovine heart mitochondrial membranes incubated with succinate and 250 µM DAB or 0.1% DMSO (control) ± 1 mg/mL PLE (mean ± SEM, *n* = 3 independent experiments). (*D*) Rat heart mitochondria were incubated with succinate (5 mM) ± DAB and H_2_O_2_ production measured by resorufin fluorescence (mean ± SEM, *n* = 4 biological replicates). (*E*) C2C12s were treated with either 250 µM DAB, 5 mM butylmalonate (BM) or 0.1% DMSO vehicle control (Vehicle) for 30 min before rapidly isolating mitochondria and measuring butylmalonate levels by LC-MS/MS (mean ± SEM, *n* = 4 (vehicle and DAB) or 5 (BM) independent experiments). (*F*) C2C12 cells were treated with DAB or vehicle (0.1% DMSO) together with 500 µM TTFA over time and succinate levels measured (mean ± SEM, *n* = 6 independent experiments). (*G*) C2C12 cells were pre-treated with DAB (15 min) prior to treatment with acidified malonate (5 mM, pH 6) for 30 min and succinate levels measured (mean ± SEM, *n* = 6 independent experiments). (*H*) C2C12 cells were pre-treated with DAB (15 min) prior to treatment with acidified succinate (5 mM, pH 6) for 30 min and succinate levels measured (mean ± SEM, *n* = 6 independent experiments). (*I*) C2C12s after siRNA knockdown of DIC, OGC, both (double) or scramble control (Scr) were treated with 500 µM TTFA + DAB or vehicle control (0.1% DMSO) for 1 h and whole cell succinate measured (mean ± SEM of *n* = 6 independent experiments). Statistical significance was assessed by Kruskal–Wallis with Dunn’s multiple comparison test (*E*—compared with control), one-way ANOVA with Dunnett’s multiple comparisons test (*G* and *H*—compared with 0 µM DAB) or two-way ANOVA with Sidak’s multiple comparisons test (*F*—DMSO vs. DAB, I—TTFA vs. TTFA + DAB).

We next assessed whether the butylmalonate delivered by DAB could affect succinate respiration by isolated heart mitochondria. DAB dose-dependently inhibited mitochondrial respiration on exogenous succinate (*Figure 5A*). DAB did not inhibit the respiration of alamethicin-permeabilized mitochondria (*Figure 5B*) or SDH activity in isolated mitochondrial membranes (*Figure 5C*). Furthermore, succinate-driven RET-ROS production was inhibited by DAB (*Figure 5D* and see Supplementary material online, *Figure S4A*) at 100-fold lower concentrations than butylmalonate itself. These results suggested butylmalonate was efficiently delivered to the mitochondrial matrix from DAB and inhibited DIC within mitochondria and thus inhibited succinate transport.

Next, to determine whether butylmalonate was delivered into the mitochondrial matrix when cells were treated with DAB, we rapidly isolated mitochondria and measured intramitochondrial butylmalonate levels by LC-MS/MS. We found that butylmalonate was efficiently delivered within the mitochondrial matrix by DAB, but not by butylmalonate alone (*Figure 5E*). To assess the effects of DAB *in cellulo*, C2C12 myoblasts were treated with the SDH inhibitor TTFA ± DAB. DAB-treated cells accumulated 2.4–3.1-fold less succinate than the control cells (*Figure 5F* and see Supplementary material online, *Figure S4B*). Butylmalonate alone had little impact on succinate accumulation with TTFA treatment, even at millimolar concentrations, further confirming the necessity of intracellular delivery using a prodrug approach (see Supplementary material online, *Figure S4C*). We confirmed this result was from the action of butylmalonate and not an off-target effect of an acetoxymethyl (AM) diester, which hydrolyses to generate formaldehyde and acetic acid,^40^ by comparison with an acetoxymethyl diester of the SDH inhibitor malonate, malonate-AM.^30^ If the decrease in TTFA-mediated succinate accumulation by DAB is due to off-target effects of the AM group hydrolysis products, malonate-AM would similarly reduce succinate accumulation. However, succinate accumulation by SDH inhibition with TTFA was unaffected by malonate-AM (see Supplementary material online, *Figure S4D*). Also, DAB led to no increased cell death during these acute incubations (see Supplementary material online, *Figure S4E*). This confirmed that the AM group hydrolysis products were not responsible for the decreased succinate accumulation by DAB. We next tested if the butylmalonate delivered by DAB could inhibit the transport of other DIC substrate molecules into mitochondria within cells. Cells were pre-treated with DAB before incubating them with acidified malonate.^27^ Malonate at low pH rapidly crosses the plasma membrane via the MCT1, and is subsequently transported into the mitochondrial matrix by the DIC,^22^ where it competitively inhibits SDH, leading to succinate accumulation under normal conditions (*Figure 5G*). With DAB present, succinate levels elevated by malonate were reduced by >70% (*Figure 5G*), despite the total cellular levels of malonate being unchanged (see Supplementary material online, *Figure S5F*), this suggests that blocking the DIC with butylmalonate from DAB prevents malonate transport into mitochondria where it would inhibit SDH. When we incubated cells with acidified succinate, which is also transported via MCT1,^11,41,42^ we found that succinate levels were further elevated in DAB-treated cells (*Figure 5H*), suggesting succinate was being prevented from entering mitochondria and being oxidized by SDH. Finally, to confirm that the target of DAB was primarily the DIC, we used DIC and OGC siRNA KD cells together with DAB. We found that DAB had little effect on succinate levels in both DIC and double KD cells, but in OGC KD cells, DAB decreased succinate accumulation (*Figure 5I*). Therefore, DAB could deliver butylmalonate effectively to cells and mitochondria and can block succinate transport in cells by inhibiting the DIC.

### 3.6 DAB disrupts succinate-driven I/R injury *in vivo*

**Figure 6:**
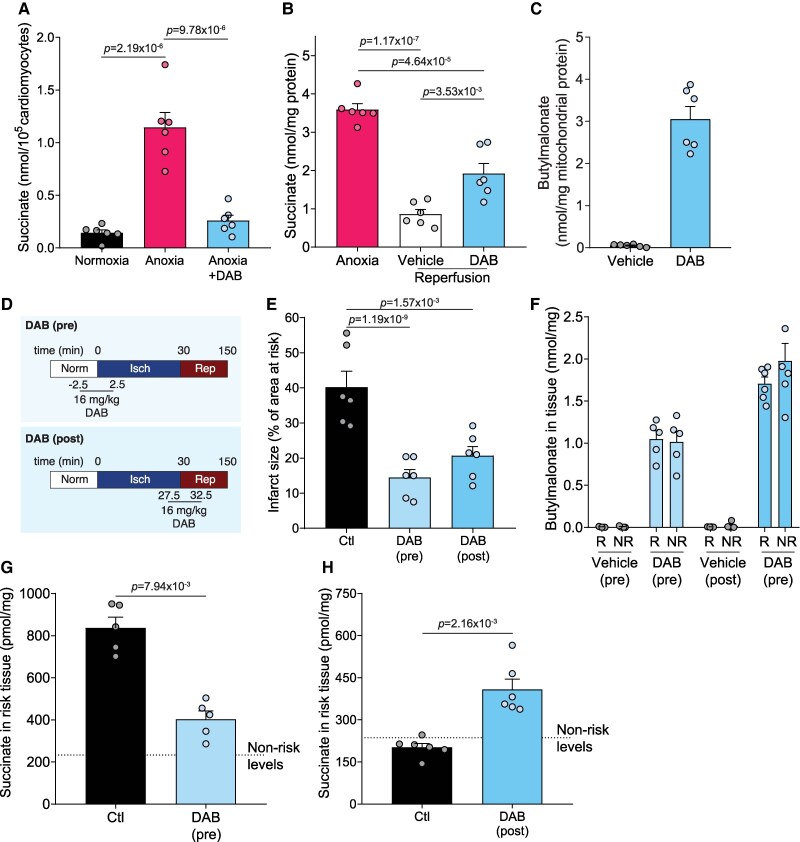
DAB is a potential therapeutic for I/R injury. (*A*) Primary adult cardiomyocytes (1 × 10^5^ cardiomyocytes/condition) were exposed to 30 min anoxia ± 250 µM DAB added 5 min into anoxia and succinate levels measured (mean ± SEM, *n* = 6 biological replicates). (*B*) C2C12s were exposed to 30 min anoxia before adding 250 µM DAB prior to 5 min reperfusion (mean ± SEM, *n* = 6 independent experiments) (*C*) C57BL/6J mice were IV injected with a bolus of DAB (16 mg/kg), after 5 min, mitochondria from hearts isolated and butylmalonate levels measured (mean ± SEM, *n* = 6 biological replicates). (*D*) Outline of *in vivo* LAD ligation experiments. (*E*) *In vivo* infarct size after 30 min LAD ligation ischaemia and 2 h reperfusion with vehicle or DAB (16 mg/kg for 5 min) starting the infusion 2.5 min before ischaemia or 2.5 min before the initiation of reperfusion (mean ± SEM, *n* = 6 biological replicates). (*F*–*H*) (*F*) Butylmalonate or (*G* and *H*) succinate levels measured in non-risk (*NR*) and at-risk (*R*) tissue either after 30 min LAD ligation ischaemia, or 30 min ischaemia and 5 min reperfusion. DAB was infused as in *D*) either before ischaemia or upon reperfusion (mean ± SEM, *n* = 5–6 biological replicates). Statistical significance was assessed by one-way ANOVA with Tukey’s multiple comparisons test (*A*), one-way ANOVA with Dunnett’s multiple comparisons test (C—compared with control) or unpaired, two-tailed Mann–Whitney *U* test (*D* and *E*).

We next tested whether DAB impacts cardiac I/R injury. First, we used isolated adult primary mouse cardiomyocytes exposed to anoxia ±DAB. As expected, anoxia alone led to ∼8-fold increase in succinate accumulation, however, when DAB was added, this largely prevented succinate accumulation (*Figure 6A*). Furthermore, DAB prevented succinate oxidation when added to cells just before reoxidation (*Figure 6B*). This suggests that DAB may have utility in understanding the contribution of succinate transport in I/R injury by either administering it before ischaemia or upon reperfusion. To explore whether DAB could be effective *in vivo*, we injected DAB intravenously into male C57BL/6J mice and 5 min after injection, rapidly retrieved (<15 s) the heart and isolated mitochondria. We measured the butylmalonate levels in the heart mitochondria and found that butylmalonate was effectively delivered to mitochondria *in vivo* (*Figure 6C*). These results suggest that DAB is a useful tool to modulate succinate transport in cardiac I/R injury *in vivo*. We next assessed the ability of DAB to prevent cardiac IR injury in an *in vivo* LAD ligation MI mouse model (*Figure 6D*). As DAB could potentially impact both ischaemic succinate accumulation and oxidation upon reperfusion, we administered it either before the induction of ischaemia or upon reperfusion (*Figure 6D*). DAB reduced infarct size after MI *in vivo* when administered either before the induction of ischaemia, or upon reperfusion (*Figure 6E* and see Supplementary material online, *Figure S5*). To understand whether this protection was due to DAB affecting succinate metabolism, butylmalonate and succinate levels were measured in the at-risk area of the heart. Butylmalonate was effectively delivered to heart tissue both when administered before ischaemia or upon reperfusion (*Figure 6F*). When DAB was infused before ischaemia, the succinate levels in the ischaemic tissue remained similar to those in healthy, non-ischaemic tissue (*Figure 6G*), suggesting DAB pre-treatment prevented ischaemic succinate accumulation. When DAB was administered upon reperfusion, the succinate levels after 5 min reperfusion remained elevated compared with both healthy or vehicle reperfused tissue (*Figure 6H*). This suggests that DAB administration upon reperfusion slows the oxidation of succinate upon reperfusion and is thereby cardioprotective. As DAB did not affect complex I activity^56^  *in vivo*, this further confirms the cardioprotective effects of DAB are from delivering butylmalonate to inhibit mitochondrial succinate transport (see Supplementary material online, *Figure S5B*).

## 4. Discussion

While succinate transport between the mitochondrial matrix and the cytosol has been implicated indirectly in I/R injury, a direct connection between this transport process and injury has not been demonstrated. Here, we found that the DIC, but not the OGC, plays a key role in modulating succinate metabolism by controlling the exchange between the mitochondrial matrix and cytosolic succinate pools.

We have shown that DAB is an effective and useful tool that can be used to understand the role of mitochondrial succinate transport in cells and *in vivo*. The AM groups in DAB are commonly used to enhance the membrane permeability and thus delivery of probes and drugs in biological systems. Furthermore, the results reported here are not due to off-target effects of AM hydrolysis, as malonate-AM did not affect succinate accumulation following SDH inhibition. DAB is the first tool that both selectively and reversibly inhibits a mitochondrial carrier *in vivo*, which opens up both new experimental possibilities as well as potential therapies targeting mitochondrial metabolite transport. As DAB releases butylmalonate in both the cytosol and mitochondrial matrix, this has the added benefit of targeting both the open matrix-facing and cytosolic-facing conformations of the mitochondrial carrier.

To estimate the relative DAB concentration in the cytosol and mitochondrial matrix, consider that 3.64 nmol butylmalonate were delivered intracellularly to ∼1 × 10^6^ C2C12 cells *in vitro*. Mitochondria isolated from C2C12s after DAB treatment contained 0.66 nmol butylmalonate in the mitochondrial matrix. Therefore, ∼18% of the total butylmalonate delivered *in vitro* is within the mitochondrial matrix. Assuming a cytosolic water volume of 5.05 µL/mg protein,^43^ mitochondria occupy 30% of the cell volume^44^ and butylmalonate quantification was from cell pellets with ∼0.35 mg protein, this equates to millimolar concentrations of butylmalonate in both the cytosol (∼2.4 mM) and the mitochondria (∼1.25 mM). Achieving these intramitochondrial butylmalonate concentrations would enable DIC inhibition in the matrix-facing conformation and thus, limit cytosol/mitochondrial transport. This prodrug approach not only facilitates the cellular entry of butylmalonate but also enables mitochondrial delivery and may lead to inhibition of the DIC in mitochondria in both conformations (see Supplementary material online, *Figure S6*), increasing its effectiveness.

DAB modulated succinate transport *in vivo* as evidenced by changes in succinate levels during ischaemia and reperfusion. Both intervening before ischaemia and upon reperfusion were protective. As DAB administered before ischaemia was also detected at the end of ischaemia, this suggests that it may have played a dual role in cardioprotection by both preventing succinate accumulation and also its oxidation by mitochondria on reperfusion. Thus, DAB may not only be useful therapeutically in MI, administered at the point of reperfusion during primary percutaneous coronary intervention, but may also be applicable in circumstances where the period of ischaemia is predictable such as organ transplantation, where DAB could be administered before ischaemia or within the organ storage solution to prevent the damaging ischaemic succinate accumulation. Furthermore, with butylmalonate retained within the tissue, when the organ is transplanted and reperfused, additional protection by blocking succinate re-entry into mitochondria may occur. As we anticipate that RET-ROS will occur within the first few minutes of reperfusion, the rapid delivery of butylmalonate from DAB is essential. We can see from cell and *in vivo* experiments that butylmalonate is very rapidly delivered and high levels of intramitochondrial butylmalonate are achieved within 5 min of administration. Therefore, it is possible to administer DAB within the relevant therapeutic window to modulate RET-ROS production in I/R injury. The impact of the breakdown products from DAB ester hydrolysis (formaldehyde and acetate) needs to be considered while the metabolism of butylmalonate itself *in vivo* is unknown, the toxicity of DAB will require further assessment for use in chronic, rather than acute, applications. Previously, highly cardioprotective agents have shown detrimental off-target effects^45,46^ and in some cases increased mortality,^47^ which precludes their clinical use. Therefore, a careful understanding of the longer-term effects of DAB on different organs will be important to determine in future studies.

Our finding that disrupting DIC activity dramatically impacts succinate accumulation following ischaemia or SDH inhibition, while OGC does not is intriguing, as both OGC and DIC have been reported to transport succinate.^22^ Direct comparisons of the transport rates of succinate by the DIC and OGC, particularly for the human proteins, are currently missing; thus, understanding the various kinetic factors at play here is incomplete. However, the difference in succinate accumulation between SDH inhibition and anoxia with OGC KD may suggest differences in the mechanism underlying succinate accumulation. OGC KD having little effect on succinate accumulation in anoxia may be due to SDH reversal being a prominent mechanism, and thereby less reliant on glutaminolysis. However, increased succinate production with SDH inhibition in OGC KD cells may be the result of less 2-oxoglutarate entering the cytosol and therefore being shunted into the production of succinate by the forward TCA cycle (see Supplementary material online, *Figure S7*). Furthermore, this suggests OGC KD may impact α-ketoglutarate-dependent hydroxylases and thus be clinically relevant in several diseases.^48–50^ Further work using stable isotope metabolite tracing will enable a more comprehensive understanding of the flux and fate of metabolites in OGC KD cells.

The roles succinate may play in health and disease have recently rapidly expanded, such as in ischaemia,^9^ exercise^42^ and inflammation.^12^ Despite this, little attention has been paid to central role of mitochondrial succinate transport in facilitating intracellular and extracellular metabolite signalling. With succinate predominantly produced in mitochondria, its movement into the cytosol is essential for its actions in other cellular compartments and also extracellularly. Our knowledge of the DIC impact on health and disease is in its infancy; however, there is evidence that the DIC may be important in white adipocyte lipolysis and the subsequent lipotoxicity associated with high-fat diet via ligation of succinate to the extracellular succinate receptor.^51^ Also, it has been suggested that DIC expression may be regulated by the circadian protein CLOCK.^52^ This has interesting implications, particularly considering the clinical evidence of increased MI severity dependent on the time of day.^53^

Furthermore, butylmalonate and other butylmalonate esters have been used previously in cancer^54,55^ and inflammation cell models,^12,13^ respectively. However, since the levels of butylmalonate were not measured in either instance, understanding their mechanism(s) of action is not possible. Robust quantification of drug levels is essential to understand whether sufficient is present to elicit a pharmacologically relevant effect and DAB should provide a more effective approach than butylmalonate or DEB in these conditions.

## 5. Conclusion

Succinate is a key driver of damage in I/R injury, due to its extensive accumulation in ischaemia and rapid oxidation on reperfusion, driving the downstream pathology. A key step in achieving high succinate levels during ischaemia is the transport of succinate from the mitochondria to the cytosol and subsequent re-entry into mitochondria predominantly via the DIC. DAB could inhibit the DIC and perturb succinate movement between the mitochondrial and cytosol pools both *in vitro* and *in vivo*, thereby affording significant cardioprotection. The role of the DIC in health and disease is in its infancy; thus, DAB may serve as a valuable tool and therapeutic in several conditions.

Translational perspective This study empirically demonstrates that DIC, but not OGC, is the primary mitochondrial transporter facilitating cytosolic succinate distribution and accumulation during ischaemia. We developed diacetoxymethyl butylmalonate (DAB), a prodrug that efficiently delivers the DIC inhibitor butylmalonate into cells and mitochondria, enabling its therapeutic application *in vivo*.

We show that modulating mitochondrial succinate transport with DAB provides robust cardioprotection against myocardial I/R injury, both when administered prior to ischaemia (thereby preventing succinate accumulation) and upon reperfusion (through attenuating succinate oxidation). This work establishes mitochondrial succinate transport via the DIC, as a direct and pharmacologically targetable mechanism for mitigating I/R injury, opening new avenues for therapeutic intervention in myocardial infarction and other conditions characterized by succinate-driven pathology.

## Supplementary Material

cvag031_Supplementary_Data

## Data Availability

Raw NMR data for DTB, DHB and DAB are available at https://dx.doi.org/10.5525/gla.researchdata.2034. Other data will be made available upon reasonable request, by contacting a corresponding author.
