# Supplementary material for: Mitochondrial succinate transport is required for cardiac ischaemia/reperfusion injury
Source: Cardiovasc Res. 2026 Jan 28;122(6):734–47. doi: 10.1093/cvr/cvag031 (PMC13123664; doi:10.1093/cvr/cvag031)
Supplement: cvag031_Supplementary_Data [file cvag031_supplementary_data.pdf]

Supplementary Material

Mitochondrial succinate transport is required for cardiac ischemia/reperfusion injury

Laura Pala[1], María Torres-López[2,3], Stuart T. Caldwell[1], Joyce Valadares[4], Emily M. Smith[4], Katherine L. Hammond[4], Olga Sauchanka[5], Jiro Abe[4], Thomas Krieg[5], Richard C. Hartley[1], Michael P. Murphy[4,5]*, Hiran A. Prag[4,5]*†

[1]School of Chemistry, University of Glasgow, Glasgow G12 8QQ, UK

[2]Institute of Biomedicine of Seville (IBiS), Hospital 'Universitario Virgen del Rocío'/CSIC/University of Seville, Seville 41013, Spain

[3]Department of Medical Physiology and Biophysics, School of Medicine, University of Seville, Seville 41009, Spain

[4]MRC Mitochondrial Biology Unit, University of Cambridge, Cambridge, CB2 0XY, UK

[5]Department of Medicine, University of Cambridge, Cambridge, CB2 0QQ, UK

†Present address: Division of Pharmacy and Optometry, Faculty of Biology, Medicine and Health, University of Manchester, Manchester, UK, M13 9PT

*Hiran A. Prag and Michael P. Murphy are joint senior authors

*Correspondence to:

Hiran A. Prag, Division of Pharmacy and Optometry, Faculty of Biology, Medicine and Health, University of Manchester, Manchester, UK, M13 9PT. Email: hiran.prag@manchester.ac.uk

Michael P. Murphy, MRC Mitochondrial Biology Unit, University of Cambridge, Cambridge, CB2 0XY, UK. Email: mpm37@cam.ac.uk

1. Supplementary Figures

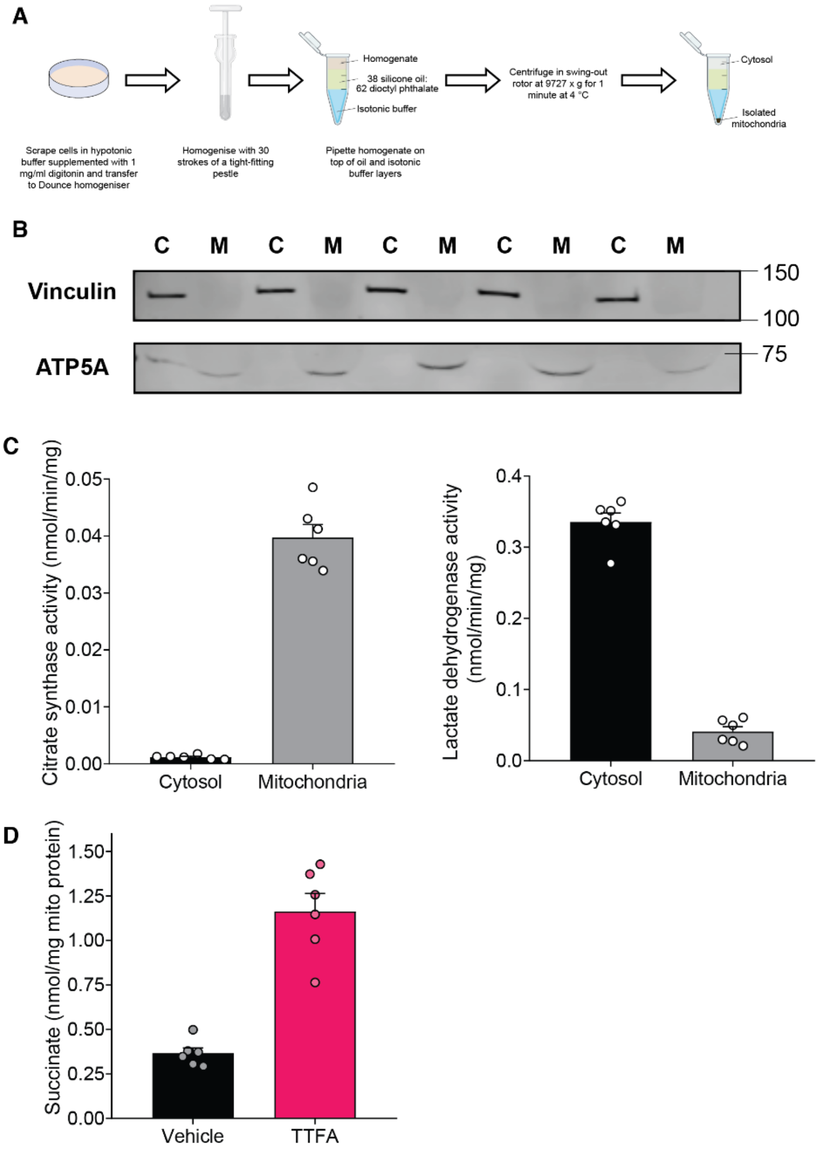

Supplementary Figure 1. Developing a rapid mitochondrial isolation method for cells. A) Schematic of the method to rapidly isolate mitochondria from minimal cell numbers. B) Representative Western blot of rapidly isolated mitochondria and cytosol fractions, blotted for vinculin and citrate synthase for the cytosol and mitochondrial fraction, respectively. C) Citrate synthase and LDH activity assays from rapidly isolated mitochondria and cytosol (mean ± SEM, n=6 independent experiments). D) C2C12 myoblasts (500,000/well) were treated with 500 μM TTFA for 1 hour, subsequently rapidly fractionated and succinate in mitochondria measured by LC-MS/MS (mean ± SEM of n=6 independent experiments).

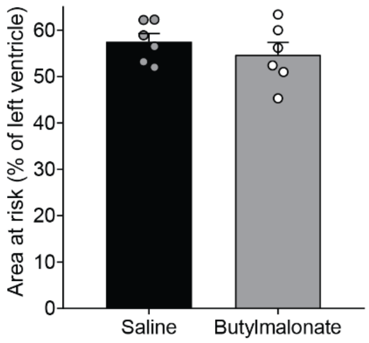

Supplementary Figure 2. LAD ligation area at risk for butylmalonate infarct. C57BL/6J mice were subjected to 30 min LAD ligation and 2 hours reperfusion ± 160 mg/kg butylmalonate infused for 10 min, starting 5 min before reperfusion. Area at risk = risk area as a percentage of the total left ventricle as determined by Evans Blue staining (mean ± SEM, n=6 biological replicates).

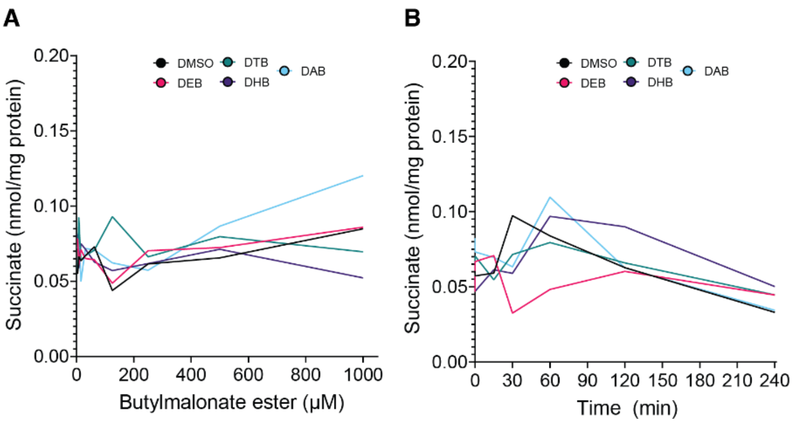

Supplementary Figure 3. Butylmalonate esters do not affect succinate levels. C2C12s were treated with A) varying concentrations or B) 250 µM of esters over time and succinate levels measured by LC-MS/MS (mean ± SEM, n=6 biological replicates). Cells treated with the SDH inhibitor positive control malonate-AM diester led to succinate levels of 6.43 ± 0.48 nmol/mg protein (mean ± SEM, n=3 biological replicates).

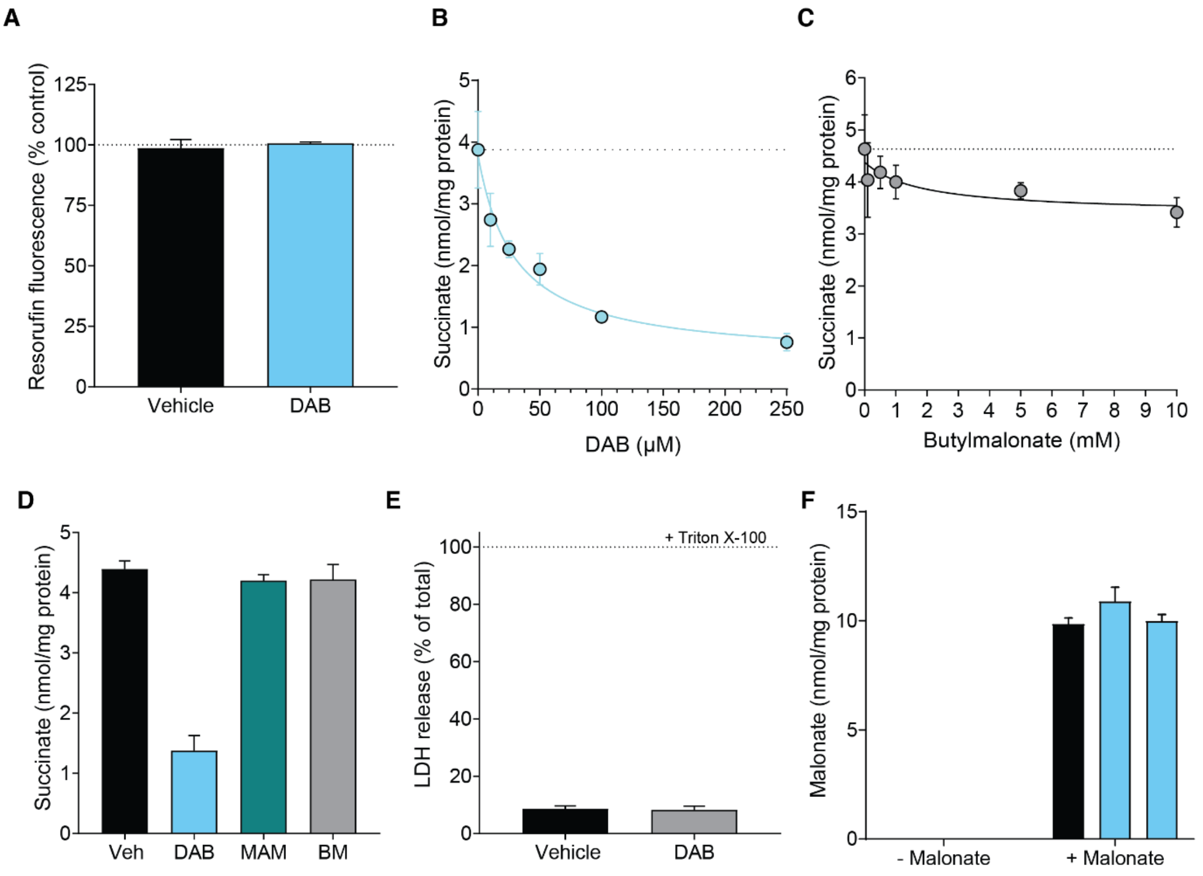

Supplementary Figure 4. A) Amplex red conversion to resorufin with hydrogen peroxide with 0.1% DMSO (Vehicle) or 250 µM DAB (mean ± SEM of resorufin fluorescence as a percentage of non-vehicle control, n=6 biological replicates. B-C) (B) DAB or (C) butylmalonate dose-dependent inhibition of succinate accumulation in TTFA-treated C2C12s. D) TTFA treated C2C12s for 15 min with 0.1% DMSO (Veh), 250 µM DAB and MAM or 5 mM butylmalonate (BM) (mean ± SEM, n=6 biological replicates). E) C2C12 LDH release after 2 hour treatment with 0.1% DMSO (Vehicle) or 250 µM DAB (mean ± SEM LDH activity as a percentage of Triton X-100 treated cells LDH activity, n=6 independent experiments). F) Malonate levels from cells treated in Fig. 5B where C2C12 cells were pre-treated with DAB (15 min) prior to treatment with acidified malonate (5 mM, pH 6) for 30 min and succinate levels measured (mean ± SEM, n=6 independent experiments).

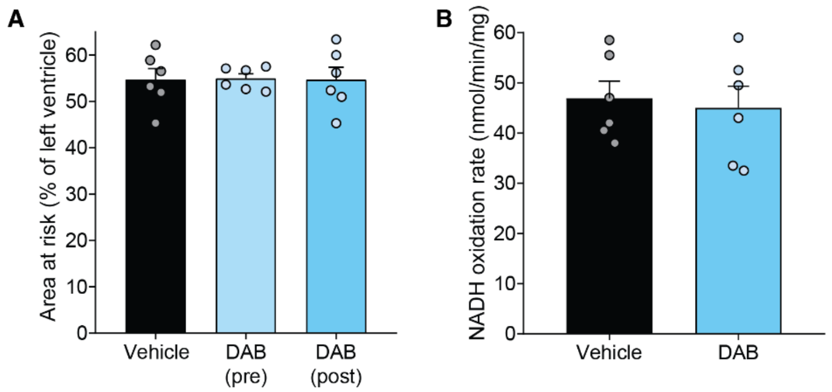

Supplementary Figure 5. A) LAD ligation area at risk for DAB infarcts. C57BL/6J mice were subjected to 30 min LAD ligation and 2 hours reperfusion ± 16 mg/kg DAB or 1% DMSO vehicle control before ischemia or reperfusion. Area at risk = risk area as a percentage of the total left ventricle as determined by Evans Blue staining (mean ± SEM, n=6 biological replicates). B) Complex I activity measured from non-risk tissue of LAD ligation as described in (A) (mean ± SEM, n=6 biological replicates).

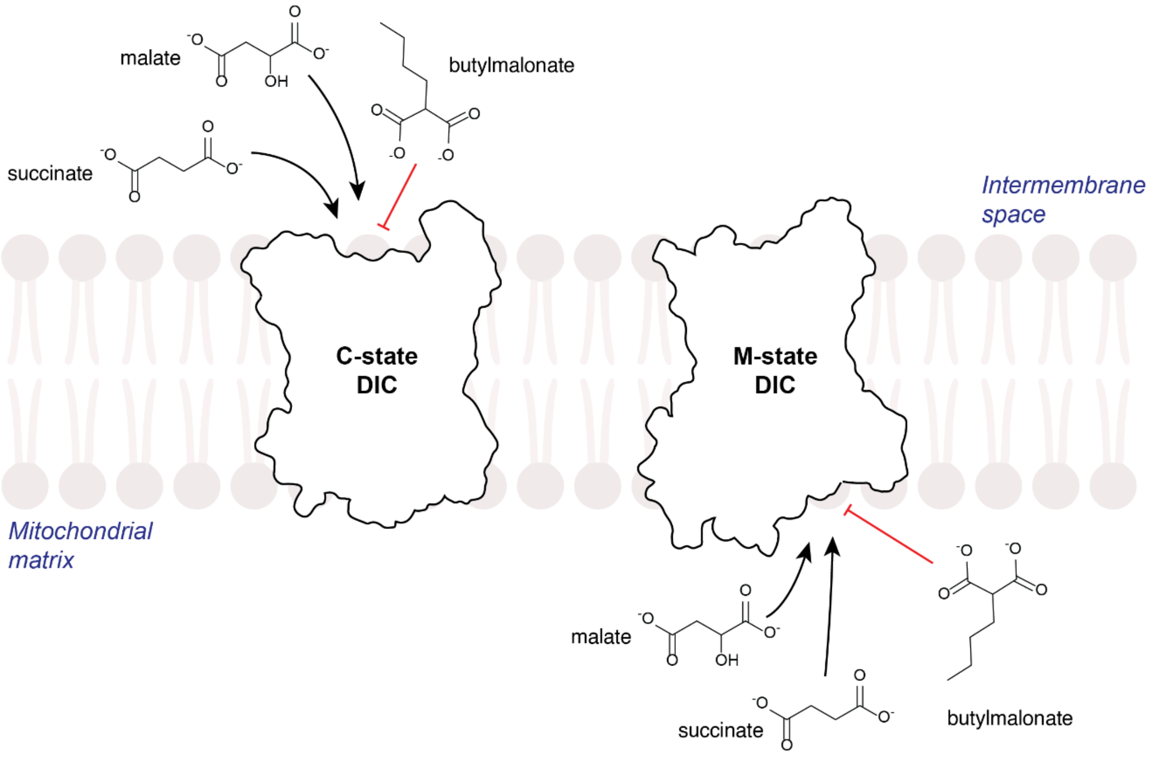

Supplementary Figure 6. Schematic of the potential dual action of butylmalonate delivered by DAB intracellularly and intramitochondrially.

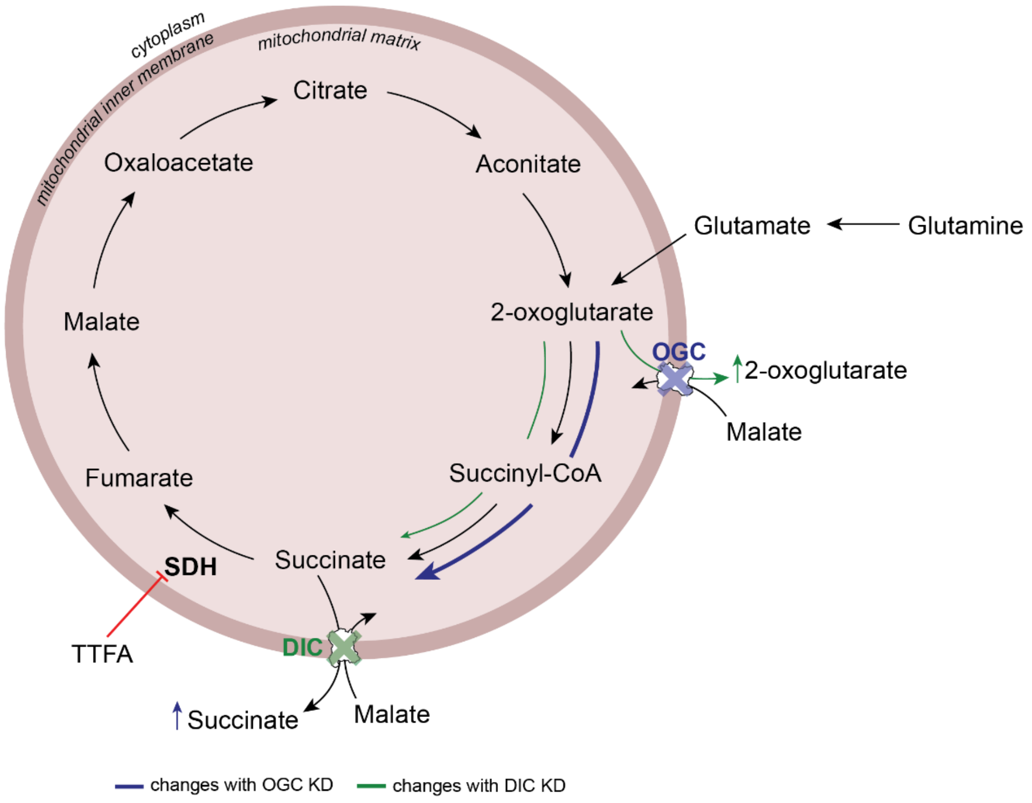

Supplementary Figure 7. Schematic of hypothesized mechanisms for succinate accumulation differences in DIC and OGC KD cells with SDH inhibited. With OGD KD, less 2-oxoglutarate may be transported out of mitochondria and thus greater flux may continue in the TCA cycle, leading to enhanced succinate accumulation upon SDH inhibition. With DIC KD, succinate may compete with 2-oxoglutarate for transport, though OGC, though it preferentially transports 2-oxoglutarate. DIC KD may favor OGC exchange of 2-oxoglutarate for malate may replenish TCA cycle flux, to bypass succinate production with SDH inhibition.

2. Supplementary Materials and Methods

2.1 Chemical Synthesis

2.1.1 Di(2,2,2-trifluoroethyl) 2-butylmalonate (DTB)

n-Butylmalonic acid (100 mg, 0.624 mmol) was dissolved in 2,2,2-trifluoroethanol (1.0 mL) under an atmosphere of argon and then catalytic amount of concentrated sulfuric acid (1 drop) was added. The reaction was heated under reflux while stirring for 16 h. The reaction was quenched with 10% aqueous sodium bicarbonate (20 mL) and the product was extracted with dichloromethane (3 × 15 mL). The combined organics were washed with brine (50 mL), dried over magnesium sulfate, filtered and the solvent was removed under reduced pressure to yield **DTB** as a yellow oil (132 mg, 0.406 mmol, 65%). ν_{max} (ATR): 2963 (CH), 2870 (CH), 1759 (C=O), 1450 (CH), 1412 (CH) cm^{-1}. δ_H (500 MHz, $CDCl_3$): 4.55 (2H, qd, *J* = 8.4, 12.7 Hz, 2 × C$\mathbf{H^A}$$H^B$$CF_3$), 4.52 (2H, qd, *J* = 8.4, 12.7 Hz, 2 × C$H^A$$\mathbf{H^B}$$CF_3$), 3.56 (1H, t, *J* = 7.5 Hz, CH), 2.02-1.93 (2H, m, CHC$\mathbf{H_2}$), 1.42-1.28 (4H, m, C$\mathbf{H_2}$C$\mathbf{H_2}$$CH_3$), 0.91 (3H, t, *J* = 7.0 Hz, CH_3). δ_C (126 MHz, $CDCl_3$): 167.33 (C), 122.72 (q, *J* = 277.2 Hz, C), 61.09 (q, *J* = 37.2 Hz, CH_2), 51.11 (CH), 29.23 (CH_2), 28.40 (CH_2), 22.28 (CH_2), 13.77 (CH_3). δ_F (471 MHz, $CDCl_3$): −73.88 (t, *J* = 8.4 Hz, CF_3). HRMS (ESI^+): 347.0681. $C_{11}H_{14}F_6NaO_4$ requires $(M+Na)^+$, 347.0688.

2.1.2 Di(1,1,1,3,3,3-hexafluoro-2-propyl) 2-butylmalonate (DHB)

n-Butylmalonic acid (500 mg, 3.12 mmol, 1 eq.) and thionyl chloride (685 µL, 6.24 mmol, 3 eq.) were dissolved in 1,1,1,3,3,3-hexafluoro-2-propanol (5.0 mL) under an atmosphere of argon while stirring and the reaction was conducted at reflux while stirring for 7 days. The solvent and residual thionyl chloride were removed under reduced pressure. The reaction mixture was then re-dissolved in dichloromethane (25 mL), washed with 5% sodium bicarbonate (4 × 50 mL) and extracted with dichloromethane (3 × 25 mL). The combined organics were washed with brine (100 mL), dried over magnesium sulfate, filtered and the solvent was removed under reduced pressure. The product was purified by passing a solution of the compound in hexane through a silica plug to yield the diester **DHB** as a yellow oil (444 mg, 0.964 mmol, 31%). R*f* [SiO_2, hexane] = 0.22. ν_{max} (ATR): 2972 (CH), 2938 (CH), 2882 (CH), 1777 (C=O), 1458 (CH) cm^{-1}. δ_H (500 MHz, $CDCl_3$): 5.78 [2H, sept, *J* = 5.9 Hz, 2 × $CH(CF_3)_2$], 3.75 (1H, t, *J* = 7.5, CHCO), 2.04 (2H, apparent q, *J* = 7.6 Hz, 2 × C$\mathbf{H_2}$CH), 1.43-1.31 (4H, m, 2 × CH_2), 0.92 (3H, t, *J* = 7.0 Hz, CH_3). δ_C (126 MHz, $CDCl_3$): 165.21

(C), 120.18 (qq, J = 2.7, 280.1 Hz, C), 67.47 (sept, J = 35.2 Hz, CH), 50.57 (CH), 28.80 (CH_2), 28.44 (CH_2), 22.14 (CH_2), 13.69 (CH_3). δ_F (471 MHz, $CDCl_3$): −73.37-−73.23 (m, CF_3^A and CF_3^B). HRMS (CI^+): 461.0605. $C_{13}H_{13}F_{12}O_4$ requires $(M+H)^+$, 461.0622.

2.1.3 Di(acetoxymethyl) 2-butylmalonate (DAB)

Acetoxymethyl bromide (202 mL, 2.06 mmol, 2.2 eq) was added to a solution of 2-*n*-butylmalonic acid (150 mg, 0.93 mmol, 1.0 eq) and diisopropylethylamine (404 mL, 2.32 mmol, 2.5 eq) in anhydrous MeCN (8.0 mL) at 0 °C under an atmosphere of argon. The solution was allowed to warm to RT then stirred overnight. The solution was concentrated under vacuum. The residue purified by column chromatography using a 12 g Agela cartridge eluting with EtOAc:Hexanes (20:80 increasing to 50:50 over 10 column volumes) to give **DAB** as a colourless oil (233 mg, 82%). δ_H (400 MHz: $CDCl_3$): 5.72 (2H, d, J = 5.7 Hz, 2 × $OC\mathbf{H^A}H^B$ or $OCH^A\mathbf{H^B}$), 5.70 (2H, d, J = 5.6 Hz, 2 × $OC\mathbf{H^A}H_B$ or $OCH^A\mathbf{H^B}$), 3.37 (1H, t, J = 7.5 Hz, C**H**CH_2), 2.07 (6H, s, 2 × $COCH_3$), 1.92-1.83 (2H, m, $CHC\mathbf{H_2}$), 1.34-1.22 (4H, m, 2 × CH_2), 0.85 (3H, t, J = 6.8 Hz, $CH_2C\mathbf{H_3}$). δ_C (101 MHz: $CDCl_3$): 169.35 (C), 167.70 (C), 79.59 (CH_2), 51.35 (CH), 29.16 (CH_2), 28.13 (CH_2), 22.23 (CH_2), 20.57 (CH_3), 13.73 (CH_3). HRMS (ESI^+): 327.1044. $C_{13}H_{20}NaO_8$ requires $(M+Na)^+$, 327.1050.

See supplementary methods figure for processed NMR data for DTB, DHB and DAB.

2.2 Hydrolysis of butylmalonate esters

Butylmalonate esters (200 µM) were incubated in KCl buffer (120 mM KCl, 10 mM HEPES, 1 mM EGTA pH 7.2, 37 °C) supplemented with porcine liver esterase (1 mg protein/ml) on a shaking heatblock (37 °C, 1000 rpm). 20 µl aliquots at various time points were extracted in 500 µl MS extraction buffer containing MS internal standard and analyzed by LC-MS/MS.

2.3 Isolation of rat heart mitochondria

Rat heart mitochondria (RHM) were isolated as described previously[30]. Briefly, rats were culled by cervical dislocation, hearts rapidly excised, washed with STEB buffer (250 mM sucrose, 5 mM Tris-Cl, 1 mM EGTA, 0.1% (w/v) BSA; pH 7.4, 4 °C) and minced finely. The tissue was homogenized using a dounce homogenizer and

centrifuged (1000 ×g, 5 min, 4 °C). The supernatant was filtered through pre-wet muslin and centrifuged (10,000 ×g, 10 min, 4 °C). The mitochondrial pellet was washed in buffer and re-centrifuged. The final mitochondrial pellet was resuspended in STE buffer (no BSA) and protein concentration measured by BCA assay (Thermo Fisher Scientific, UK).

2.4 Complex II + III activity assay

Bovine heart mitochondrial membranes (BHMMs; 80 μg protein/ml; prepared as described previously[56]) were incubated in KPi buffer (34.8 mM K_2HPO_4, 15.2 mM KH_2PO_4, 1 mM EDTA, 3 mM KCN, 4 μg/ml rotenone, pH 7.4, 4 °C) supplemented with 0.2, 1 or 5 mM succinate and with or without butylmalonate or DAB in a 96-well plate. 30 μM oxidized cytochrome c was added and the reduction of cytochrome c followed spectrophotometrically at 550 nm (20-s intervals for 5 min, 30 °C; Spectramax Plus 384, Molecular Devices, UK).

2.5 Complex I activity assay

KPi buffer containing 0.2 mM KCN and 0.3 μM antimycin A was aliquoted into an appropriate number of wells on a 96-well plate on wet ice. dQ (0.2 mM final) was added to each well as well as EtOH or Piericidin A (1 μM final), a complex I inhibitor used as negative control. Tissue homogenate was diluted to 10 μg protein per 100 μL in KPi buffer containing 0.3% CHAPs and 0.3% Asolectin and incubated on wet ice for 5 minutes. 100 μL of tissue homogenate was added to two wells containing EtOH or Piericidin A. The assay was initiated with the addition of NADH (0.2 mM final) and absorbance measured at 340 nm and 380 nm in 12 second intervals for 30 minutes at 30°C using a UV-Vis microplate reader (CLARIOstar Plus, BMG Labtech). To quantify the rate of NADH oxidation, the absorbance at 340 nm was subtracted from that at 380 nm and the maximum gradient of the curve was calculated using MARs data analysis software. The maximum gradient of Piericidin A treated samples was subtracted from that of the EtOH-treated samples. Then the concentration of NADH was determined by the Beer-Lambert law using an extinction coefficient of ε=340-380 4.81 $mM^{-1}cm^{-1}$.

2.6 Butylmalonate delivery to rat heart mitochondria

Isolated rat heart mitochondria (1 mg protein/ml) were incubated in KCl buffer (pH 7.4; 37 °C) supplemented with succinate (5 mM) and rotenone (4 μg/ml) and butylmalonate esters (250 μM) for 5 min before pelleting mitochondria (10,000×g, 5 min, 4 °C), washing the pellet and recentrifuging before pellets were extracted with 500 μl MS extraction buffer containing MS internal standard and analyzed by LC-MS/MS.

2.7 Measurement of ROS production by RET

ROS production by RET in isolated heart mitochondria was measured as described previously[11]. Isolated RHM (70 μg protein/well) were incubated in KCl buffer (pH 7.2, 37 °C) supplemented with Amplex Red (12.5 μM; Invitrogen, Thermo Fisher Scientific), horseradish peroxidase (20 μg/ml), BSA (200 μg/ml), superoxide dismutase (40 μg/ml) and either butylmalonate, butylmalonate ester prodrugs or vehicle control and RET initiated with 5 mM succinate. Resorufin fluorescence was detected by a fluorometric plate reader (λex = 570 nm and λem = 585; SpectraMax Gemini XS; Molecular Devices) at 37 °C and calibrated using a hydrogen peroxide standard curve (ε=46.6 M^{-1} cm^{-1} at 240 nm).

2.8 LDH cell death assay

The toxicity of DAB was assessed as described in detail previously[30], using an LDH release cytotoxicity detection kit (Roche, UK). 10,000 C2C12 cells/well in a 96-well plate were treated with 0.1% DMSO or 250 μM DAB for 2 hours in phenol red and FBS-free DMEM media. Cells were centrifuged (250 x g, 10 min, 4 °C) and 100 μl supernatant measured for LDH activity according to manufacturer's instructions. Activity of released LDH was normalized to total LDH from cells treated with 1% Triton X-100.

3. Supplementary Methods Figure

Processed NMR spectra of all synthesized butylmalonate compounds are provided.

3.1 DTB processed NMR Spectra

DTB, LP071, ^{1}H NMR 500 MHz

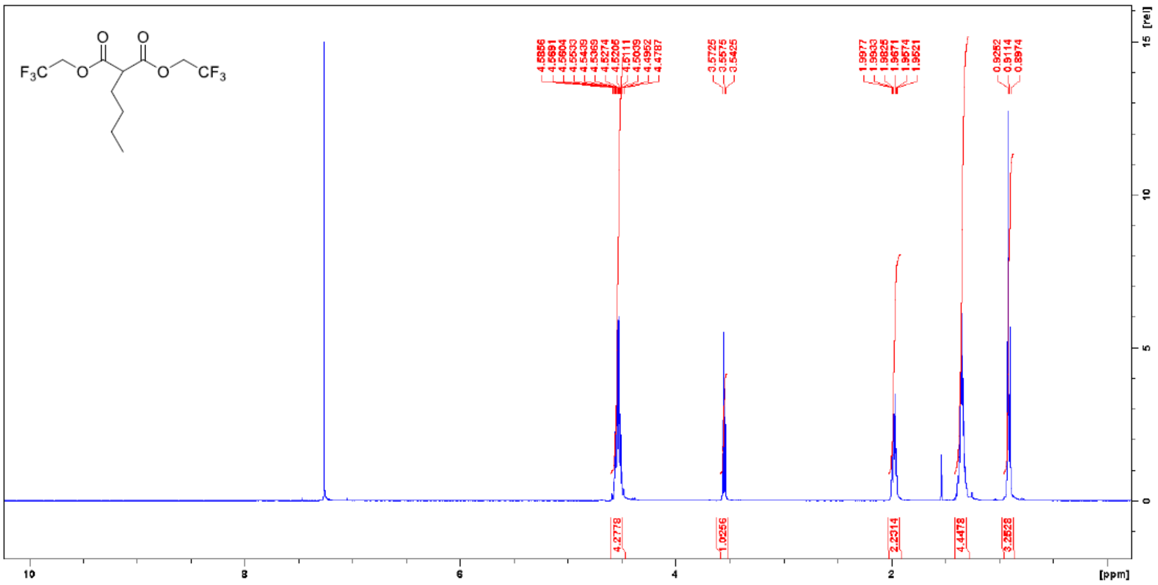

DTB, LP071, ^{13}C NMR 126 MHz

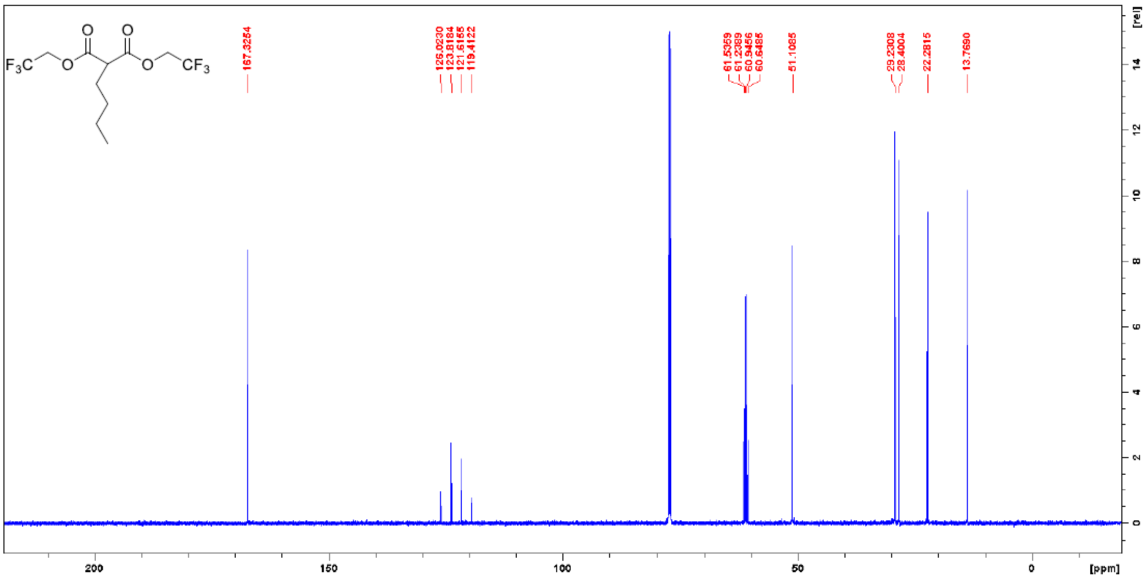

DTB, LP071, ^{19}F NMR 471 MHz

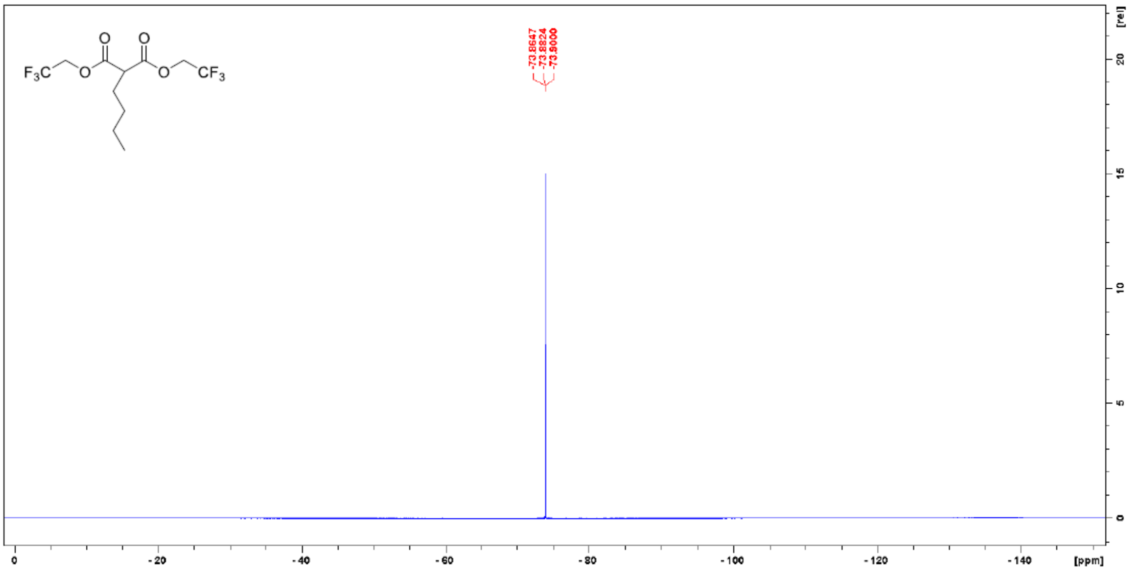

3.2 DHB processed NMR Spectra

DHB, LP095, ^{1}H NMR 500 MHz

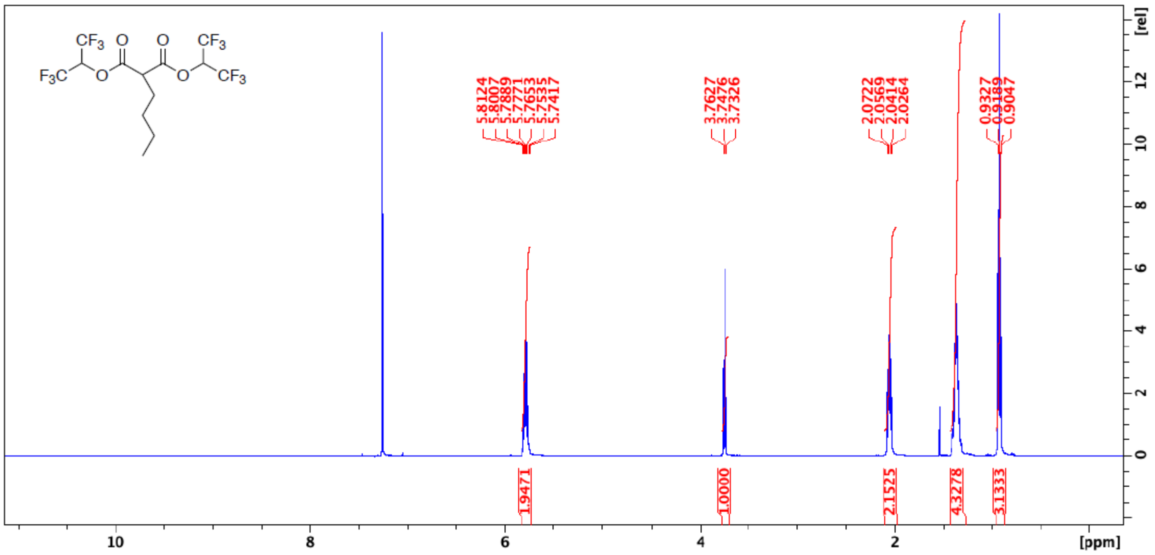

DHB LP095, ^{13}C NMR 126 MHz

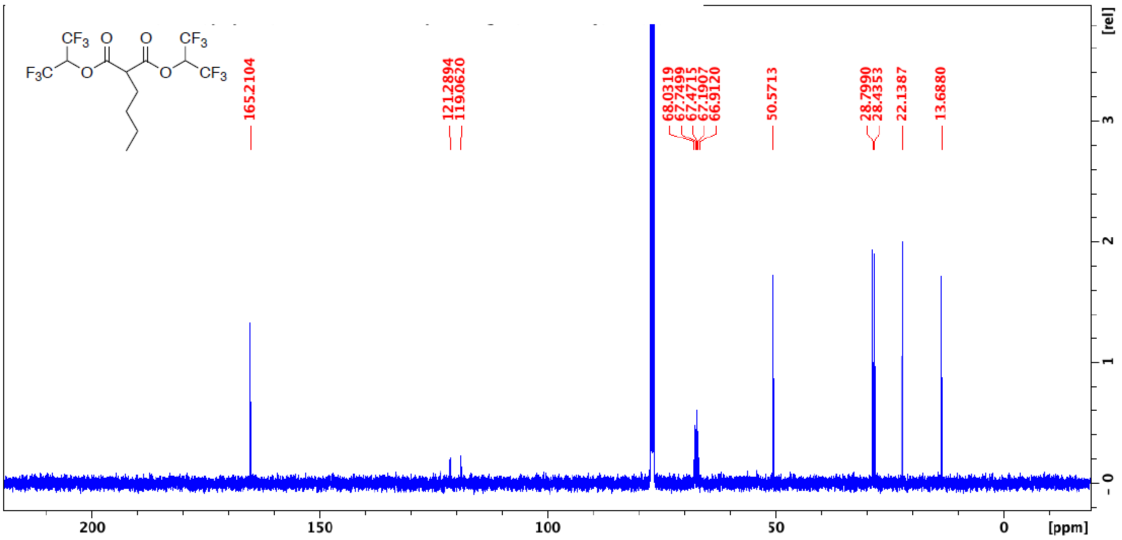

DHB LP095, ^{19}F NMR 471 MHz

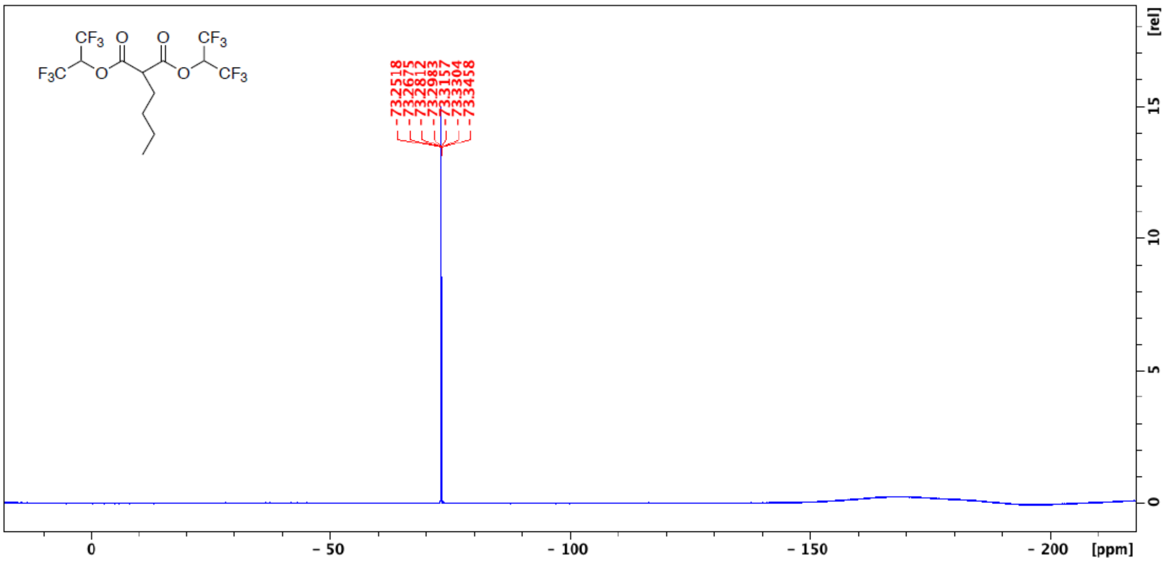

3.3 DAB processed NMR Spectra

DAB SC-6-38 ^{1}H NMR 400 MHz

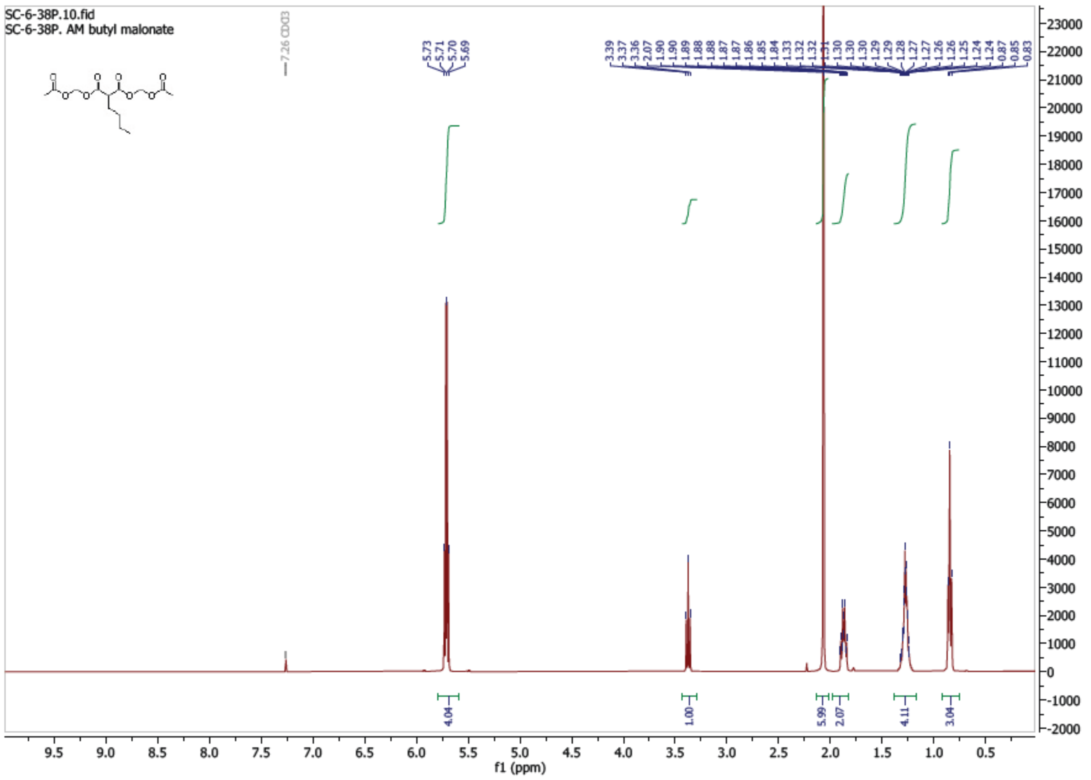

DAB SC-6-38 ^{13}C NMR 101 MHz

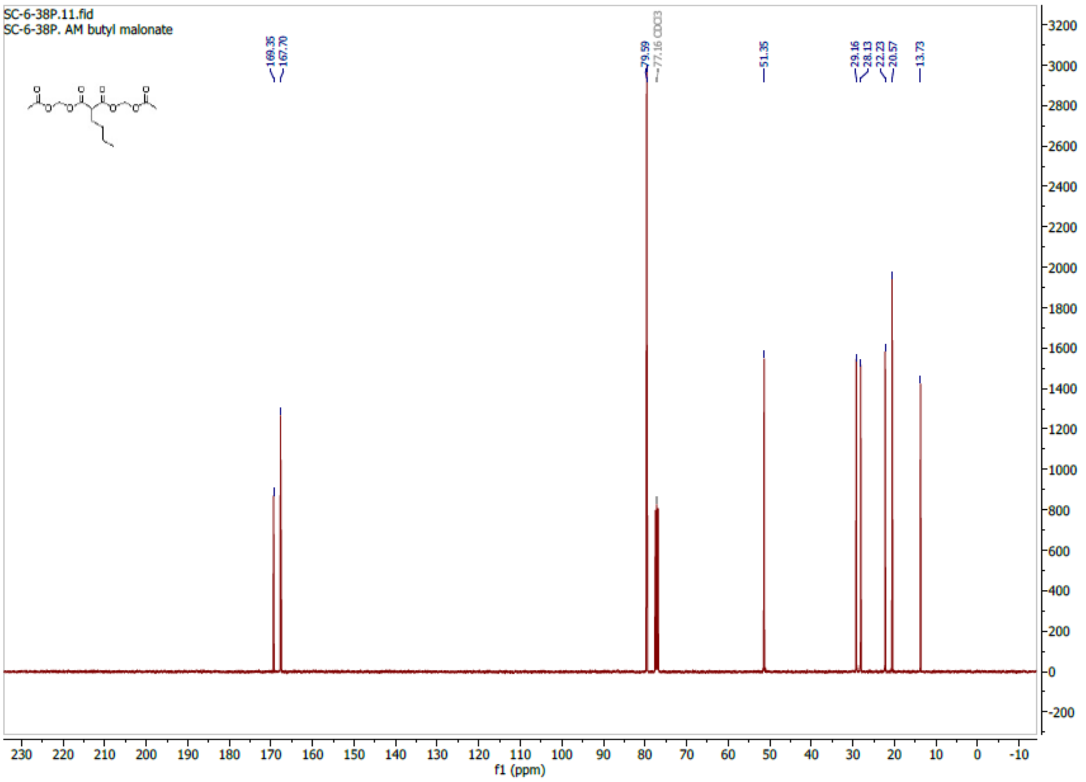

4. Supplementary Resource Table

**Description**	**Source / Repository**	**Persistent ID / URL**
Sodium malonate dibasic	Merck	63409
Carbonyl Cyanide p-(Trifluoromethoxy)phenylhydrazone (FCCP)	Merck	C2920
Oligomycin from *Streptomycin diastatochromogenes*	Merck	O4876
Rotenone	Santa Cruz	sc-203242
Antimycin A from *Streptomyces sp.*	Merck	A8674
AZD3965	Cayman Chemical	19912
AR-C141990 hydrochloride	Biotechne (Tocris)	5658
Lipofectamine RNAiMAX	Invitrogen	13778150
DMEM (high glucose, GlutaMAX, pyruvate)	Gibco	31966047
Fetal Bovine Serum	Gibco	10270106
Opti-MEM	Gibco	31985070
[$^{13}C_3$]-malonate	Merck	490202
[$^{13}C_4$]-succinate	Merck	491985
DMEM (high glucose, GlutaMAX, pyruvate)	Gibco	31966047
Silencer Select *slc25a10* siRNA mouse	Thermo Fisher Scientific	siRNA#: s211655 cat#: 4390771
Silencer Select *slc25a11* siRNA mouse	Thermo Fisher Scientific	siRNA#: s85784 cat#: 4390771
Silencer Select Negative Control No. 1 siRNA	Thermo Fisher Scientific	cat#: 4390843
PowerUp SYBR Green Master Mix for qPCR	Applied Biosystems	A25742
Butylmalonic acid	Tokyo Chemical Industry UK Ltd.	B4414
